# Neutrophil-specific deletion of Syk results in recruitment-independent stabilization of the barrier and a long-term improvement in cognitive function after traumatic injury to the developing brain

**DOI:** 10.1016/j.nbd.2021.105430

**Published:** 2021-06-19

**Authors:** Alpa Trivedi, Kayleen G. Tercovich, Amy Jo Casbon, Jacob Raber, Clifford Lowell, Linda J. Noble-Haeusslein

**Affiliations:** a Departments of Laboratory Medicine, University of California San Francisco, San Francisco, CA 94143, USA; b Departments of Neurological Surgery, University of California San Francisco, San Francisco, CA 94143, USA; c Departments of Anatomy, University of California San Francisco, San Francisco, CA 94143, USA; d Departments of Behavioral Neuroscience, Neurology, and Radiation Medicine, ONPRC, Oregon Health & Science University, Portland, OR 97239, USA; e Division of Neuroscience, ONPRC, Oregon Health & Science University, Portland, OR 97239, USA; f Departments of Neurology and Psychology, The Dell Medical School and the College of Liberal Arts, University of Texas, Austin, TX 78712, USA

**Keywords:** Neutrophils, Spleen tyrosine kinase, Oxidative stress, Barrier, Learning, Memory, Anxiety

## Abstract

While traumatic brain injury (TBI) is the leading cause of death and disability in children, we have yet to identify those pathogenic events that determine the extent of recovery. Neutrophils are best known as “first responders” to sites of infection and trauma where they become fully activated, killing pathogens via proteases that are released during degranulation. However, this activational state may generate substantial toxicity in the young brain after TBI that is partially due to developmentally regulated inadequate antioxidant reserves. Neutrophil degranulation is triggered via a downstream signaling pathway that is dependent on spleen tyrosine kinase (Syk). To test the hypothesis that the activational state of neutrophils is a determinant of early pathogenesis and long-term recovery, we compared young, brain-injured conditional knockouts of Syk (*syk*^*f/f*^*MRP8-cre*^+^) to congenic littermates (*syk*^*f/f*^). Based upon flow cytometry, there was an extended recruitment of distinct leukocyte subsets, including Ly6G^+^/Ly6C^−^ and Ly6G^+^/Ly6C^int^, over the first several weeks post-injury which was similar between genotypes. Subsequent assessment of the acutely injured brain revealed a reduction in blood-brain barrier disruption to both high and low molecular weight dextrans and reactive oxygen species in *syk*^*f/f*^*MRP8-cre*^+^ mice compared to congenic littermates, and this was associated with greater preservation of claudin 5 and neuronal integrity, as determined by Western blot analyses. At adulthood, motor learning was less affected in brain-injured *syk*^*f/f*^*MRP8-cre*^+^ mice as compared to *syk*^*f/f*^ mice. Performance in the Morris Water Maze revealed a robust improvement in hippocampal-dependent acquisition and short and long-term spatial memory retention in *syk*^*f/f*^*MRP8-cre*^+^ mice. Subsequent analyses of swim path lengths during hidden platform training and probe trials showed greater thigmotaxis in brain-injured *syk*^*f/f*^ mice than sham *syk*^*f/f*^ mice and injured *syk*^*f/f*^*MRP8-cre*^+^ mice. Our results establish the first mechanistic link between the activation state of neutrophils and long-term functional recovery after traumatic injury to the developing brain. These results also highlight Syk kinase as a novel therapeutic target that could be further developed for the brain-injured child.

## 1. Introduction

Traumatic brain injury (TBI) is one of the major causes of mortality and morbidity in children (Dewan et al., 2016; Thurman, 2016). Moreover, brain-injured children show poorer neurological outcomes as compared to adults that sustain a similar degree of injury severity (Giza et al., 2009; Giza and Prins, 2006). The intensive metabolic, cellular and vascular demands of the developing brain and low antioxidant reserves render the young brain more sensitive to reduced blood flow (Obermeier et al., 2013; Toga et al., 2006). The long-term consequences of an early age TBI include cognitive impairments and emotional disorders (Ichkova et al., 2017), poor academic and work performance and social dysfunction (Babikian et al., 2015; Ryan et al., 2016). Despite the importance of these clinical findings, there has been little progress in defining those early pathophysiological events that are key determinants of long-term neurological recovery.

Neutrophils are likely key initiators of secondary pathogenesis in the injured brain; they are critical innate immune effectors and “first responders” after TBI (Simon et al., 2017). When activated, they release potent premade granular content including oxidants, proteinases, cationic peptides, chemokines, cytokines, and reactive oxygen species (ROS) that are necessary to kill invading pathogens. However, in the case of sterile injuries such as TBIs (Jassam et al., 2017), unregulated release of these granular contents into the parenchyma promotes secondary tissue damage.

Neutrophils are activated by a diverse repertoire of receptors including the leukocyte-specific CD18 integrins. The binding of ligands to integrins is not only necessary for neutrophil adhesion and migration to the site of trauma or inflammation (Etzioni et al., 1999) but also results in activation of Src (non-receptor tyrosine kinase expressed by the *Src* gene) and Syk (spleen tyrosine kinase) tyrosine kinases (Mocsai et al., 2006). Activation of these kinases results in the subsequent secretion of granular components, release of cytokines, and generation of ROS (Lowell, 2011). Blockade/loss of this signaling pathway reduces pathogenesis in a variety of disease models including, thrombohemorrhagic vasculitis (Hirahashi et al., 2006), arthritis (Elliott et al., 2011; Nemeth et al., 2020) and *S aureus* and *Escherichia coli* infections (Van Ziffle and Lowell, 2009).

Here we consider the neutrophil-specific Syk signaling pathway in the context of the injured developing brain, where early secondary pathogenesis is superimposed on developmental processes. Disruption of the blood-brain barrier (BBB) occurs within the first day after an early age TBI and coincides with the prominent recruitment of neutrophils to the damaged cortical mantle as well as subcortical structures (Beaumont et al., 2006; Claus et al., 2010; Semple et al., 2015b; Tsuru-Aoyagi et al., 2009). Neutrophils are capable of disrupting the barrier through processes that include release of their granular content during transcellular migration (Catz and McLeish, 2020). Disruption of the BBB, in turn, is linked to adverse pro-inflammatory events (Erickson and Banks, 2013) and contributes to the secondary pathogenesis as seen in a variety of neurodegenerative diseases (Bell et al., 2012; Najjar et al., 2017; Rosenberg, 2012; Zlokovic, 2008). In addition, these acute vascular changes have been suggested to play a role in long-term cognitive and emotional dysfunction (Ichkova et al., 2017; Jullienne et al., 2016; Jullienne et al., 2014; Pop and Badaut, 2011; Pop et al., 2013).

In this study, we assessed the extent to which the activation state of neutrophils is a determinant of early secondary pathogenesis and long-term behavioral deficits after traumatic injury to the developing brain. Young brain-injured mice, with a neutrophil specific deletion of *syk* showed an acute reduction in ROS, coincident with stabilization of the BBB, including tight junction-related proteins, and greater preservation of neurons. Despite this evidence of BBB stabilization, the magnitude and temporal pattern of recruitment of these leukocytes remained unchanged, suggesting alternative pathways, independent of endothelial paracellular transport, for recruitment into the injured brain. Importantly, deletion of *syk* resulted in improved spatial learning and memory retention at adulthood after an early age brain injury. These data establish the first mechanistic link between activated neutrophils in the acutely injured developing brain and the emergence of long-term cognitive deficits. Understanding the unique pathophysiological mechanisms underlying secondary damage is central to the development of new therapeutics that are specifically tailored to the brain-injured child.

## 2. Materials and methods

### 2.1. Animals

*syk*^*f/f*^*MRP8-cre*^+^ mice were generated with conditional deletion of the *syk* gene under control of the MRP8 promoter, driving neutrophil specific excision of the gene (Van Ziffle and Lowell, 2009). Their littermate controls, *syk*^*f/f*^, have an insertion of loxP sites flagging the gene of interest. The *syk*^*f/f*^*MRP8-cre*^+^ and *syk*^*f/f*^ mice are viable and fertile and produce litters at normal Mendelian ratios. We have confirmed the efficiency and specificity of this cre-based deletion of syk (Abram et al., 2014).

Animals were housed in a specific pathogen-free facility at the UCSF Parnassus campus. Standard rodent chow and water were available ad libitum, and the housing room was maintained on an automated 12 h light/dark cycle at approximately 20 °C. All surgical and behavioral procedures were conducted in accordance with the NIH Guidelines for the Care and Use of Laboratory Animals, and approved by the UCSF Institutional Animal Care and Use Committee. A total of 184 male mice were used for this study, and randomization and blinding were applied to all experiments (Supplemental Fig. 1).

### 2.2. Controlled cortical impact model

Male mice were studied after traumatic brain injury (TBI) at postnatal day 21 (p21). This age approximates that of a toddler-aged child, based upon the structural, biochemical and behavioral characteristics of this age (Semple et al., 2013). Pups were weaned at p21 and anesthetized with 1.25% 2,2,2-tribromoethanol (Avertin; Sigma-Aldrich, St. Louis, MO) in isotonic saline via intraperitoneal administration of 0.03 ml/g body weight. After craniotomy, mice were subjected to a controlled cortical impact injury at 4.5 m/s velocity and 1.73 mm depth of penetration, for a sustained depression of 150 ms, using a 3.0 mm convex impactor tip (Semple et al., 2015b). Mice remained positioned on a water-circulating heating pad throughout surgery and recovery. Following impact, the scalp was closed with sutures and each animal administered 0.5 ml of isotonic saline subcutaneously to prevent post-operative dehydration. Sham-operated mice underwent identical surgical procedures, including craniotomy, without receiving the cortical impact. Mice were weighed post-surgery at days 1, 3, and weekly thereafter. Criteria for euthanasia was a weight loss greater than 15%; however, no mice met this criterion.

### 2.3. Flow cytometry

Brain punches, encompassing the injury site in the ipsilateral hemisphere (entire dorso-ventral region) and a similar size and location on the contralateral hemisphere, were homogenized in cell culture medium, RPMI-without phenol red. The homogenates were strained through a 40 um Nylon cell strainer (BD Falcon). Samples were lysed to remove red blood cells (BD PharmLyse^™^ lysing buffer, BD Biosciences, San Jose, CA), stained with a live/dead marker, Ghost Dye 780 (1:1000, TONBO Bioscience, San Diego, CA), followed by a wash and incubation with anti-mouse CD16/32 blocking antibody, purified clone 93 (1:100, eBioscience, San Diego, CA). After blocking, cells were washed and stained with a neutrophil panel of markers including: Violet Fluor 450 anti-mouse CD45 (1:100, TONBO Bioscience), FITC anti-mouse CD11b (1:200, eBioscience), Brilliant Violet 570 anti-mouse Ly6G, clone 1A8 (1:100, BioLegend, San Diego, CA), and PerCP/Cy5.5 anti-mouse Ly6C (1:600, BioLegend). An intracellular staining protocol was used for Alexa Fluor 647 CD206 (1:100, BioLegend), following recommendations by the vendor. Spectral compensation was achieved by using compensation beads conjugated to the above mentioned antibodies and following a vendor recommended protocol. Data for flow cytometry were acquired on a BD LSR Fortessa cytometer (BD Immunocytometry Systems) equipped with 355 nm, 405 nm, 488 nm, 561 nm, and 640 nm excitation lasers. Data were collected and analyzed using BD FACS Diva Software (BD Biosciences) and FlowJo software (FLOWJO, Ashland, OR). All analyses of flow cytometry were conducted in the Parnassus Flow Cytometry Core Facility at UCSF.

### 2.4. Histological analysis

#### 2.4.1. Tissue collection

At either 24 h post-injury or upon completion of behavioral assessments at ~2 months post-injury, anesthetized mice were perfused transcardially with ice-cold 0.1 M PBS followed by 4% PFA in 0.1 M PBS. Brains were post-fixed overnight in 4% PFA, transferred into a 30% sucrose solution for 72 h, and then embedded in Neg50^™^. Serial coronal sections, spanning the entire cortex, were cut at 20 μm or 40 μm for time points of 24 h or 2 months post-injury, respectively.

#### 2.4.2. Arginase 1 and 1A8 colocalization

Arginase 1 was used as a marker for non-classical immune cells (Fridlender et al., 2009). Non-specific binding was blocked by application of a 10% normal donkey serum solution containing 1% bovine serum albumin in PBS for 1 h, followed by overnight incubation with goat anti arginase 1 (1:50, Santa Cruz Biotechnology) and rat anti Ly6G (1:50). Positive immunolabeling was detected using Alexa fluor 488 anti-goat IgG and Alexa fluor 594 anti-rat IgG respectively. Images were captured on Zeiss confocal microscope and maximum projection images generated.

#### 2.4.3. Measurement of BBB permeability

Anesthetized mice were injected with tetramethylrhodamine (TMR) conjugated 70 kDa dextran (1 mg/10 g mouse, Invitrogen-Thermo Fisher Scientific, Waltham, MA) and fluorescein (FITC) conjugated 500 kDa dextran (1 mg/10 g mouse, Invitrogen-Thermo Fisher Scientific) into the retro orbital socket. One hour later, animals were perfused with 4% PFA in 0.1 M PBS. Brains were post-fixed overnight in 4% PFA and then transferred into a 30% sucrose solution for 72 h before being embedded in Neg50^™^ (Richard-Allan Scientific-Thermo Fisher Scientific). Serial coronal sections, spanning the entire cortex, were cut at 20 μm.

Images of sections were captured using a Nikon Eclipse 80*i* fluorescent microscope equipped with SPOT^™^ Imaging Solutions software (SPOT imaging). Six evenly spaced sections per brain (240 μm interval) were examined across the lesion starting at Bregma −1.46 mm and ending at Bregma −3.08 mm. Three to six images, encompassing the dorsal cortex and hippocampus on both the ipsilateral and contralateral hemispheres, were captured. Quantification was restricted to the region immediately adjacent to the site of impact in order to avoid vasculature that had been exposed to the direct mechanical injury.

To avoid a confound resulting from fluorescent tracers that had remained in the vasculature after perfusion fixation, images were first manually thresholded, using MetaMorph image analysis software (Molecular Devices) to detect dextrans within blood vessels. Three images per genotype (injured mice) were selected to establish the baseline vascular intensity threshold. This value was averaged and to reduce subjectivity, the same average threshold value was then applied to each image (both sham as well as injured) to measure the vascular fluorescent intensity. We next measured the intensity of fluorescence without thresholding, which represented total tissue fluorescence. BBB leakage was then defined as total tissue fluorescence - blood vessel intensity.

### 2.5. Western blot analyses

Samples, prepared from the ipsilateral cortex at the site of injury and a similar location in the contralateral cortex, were homogenized in Glo Lysis buffer (Promega, Madison, WI), containing a Complete protease inhibitor cocktail tablet (Roche Applied Sciences, Indianapolis, IN), and protein concentrations were determined by a BCA protein assay (Pierce-Thermo Fisher Scientific). Samples (50 μg protein) were boiled in Laemmli buffer containing 5% β-mercaptoethanol, then separated in a 10% SDS-polyacrylamide gel and transferred onto an activated PVDF Immobilon-FL membrane (Millipore, Bellerica, MA). Membranes were incubated with Odyssey blocking buffer (Li-Cor Biosciences, Lincoln, NE) for 1 h, followed by overnight incubation at 4 °C with antibodies against NeuN (monoclonal anti-mouse, 1:5000, clone A60; Chemicon/Millipore), Claudin-5 and β-actin (polyclonal rabbit or mouse monoclonal, 1:20,000; Sigma Aldrich). Secondary antibodies were next applied for 1 h (IRDye800-conjuated goat anti-mouse IgG at 1:20,000, and IRDye680-conjugated goat anti-rabbit IgG at 1:20,000; Li-Cor Biosciences). Immunoreactivity was detected with an Odyssey Infrared Scanner at wavelengths of 700 and 800 nm, and analyses of the bands were performed using Odyssey v1.2 software. We measured protein levels in each sample and equivalent amounts (50 μg) were loaded per lane. As beta actin levels were unchanged between any of the groups (sham versus injury and also between the two injured genotypes), the intensity of protein bands (claudin-5 or NeuN) was expressed relative to β-actin, which served as a loading control.

### 2.6. Detection of ROS by flow cytometry

Brain tissue was collected, as described above, from naïve and injured mice at 1 day post-injury. Homogenates were strained through a 40um Nylon cell strainer (BD Falcon). Samples were lysed to remove red blood cells (BD PharmLyse^™^ lysing buffer, BD Biosciences, San Jose, CA) and stained with a live/dead marker, Ghost Dye 780 (1:1000, TONBO Bioscience, San Diego, CA) and CellROX^®^ Deep Red dye (Molecular Probes) to detect reactive oxygen species (ROS). Positive controls were generated by mixing contralateral brain punches with blood and an inducer of ROS that was incubated with the CellROX dye. Negative controls were generated in the same manner, with the exception that the incubation in dye was omitted. All samples were analyzed by flow cytometry.

### 2.7. Volumetric measurements of the cortex and hippocampus

To estimate cortical and hippocampal volumes, 40 μm coronal sections, collected at ~2 months post-injury, were stained with cresyl violet. The unbiased Cavalieri method was performed with StereoInvestigator software (MicroBrightField v10.21.1), using a Nikon E600W microscope configured with a motorized stage, MAC 5000 controller and Retiga 2000R color digital camera (QImaging). Spanning Bregma 1.5 to −3.8 mm, 11–15 sections from the cortex and 4–8 sections from the hippocampus were assessed per brain. We used a sampling interval of 8 and a grid size of either 200 μm or 75 μm for the cortex and hippocampus, respectively. Measurements were confined to the dorsal hemispheres as previously described (Semple et al., 2015a; Semple et al., 2015b) using 2× and 4× objectives for the cortex and hippocampus, respectively. The Gundersen mean coefficient of error (m = 1) was maintained ≤0.02 for cortical volume estimates and ≤ 0.05 for the hippocampus and dentate gyrus (DG). Group means are expressed as estimated volume (mm^3^). Sections were excluded from stereological analyses if there was evidence of poor tissue integrity, folding, or incomplete staining. In these cases, the section was deemed to be ‘missing’ and estimation for this section was calculated from the StereoInvestigator program based upon the adjacent sections. This was permitted for a maximum of 2 non-adjacent sections per brain. Final group sizes for this analysis were therefore a subset of those used for behavioral assessment at this chronic time point (*n* = 11–12 for cortex; n = 11–13 for the hippocampus including the dentate gyrus).

### 2.8. Behavioral assessments

All behavioral tests were performed by researchers blinded to injury and genotype and were conducted within the Neurobehavioral Core for Rehabilitation Research at UCSF. Behavioral testing commenced at adulthood (2 months after injury or sham surgery). These experiments were conducted across four independent cohorts and 4 randomized groups (sham-*syk*^*f/f*^, sham- *syk*^*f/f*^*MRP8-cre*^+^, TBI-*syk*^*f/f*^, and TBI- *syk*^*f/f*^*MRP8-cre*^+^).

An extensive battery of behavioral assessments was performed in the following order: open field, rotarod, elevated plus maze, resident-intruder, and Morris water maze (MWM). Mice were habituated to individual clean cages 24 h prior to testing. Equipment, used for testing, was cleaned with 10% bleach (0.5% sodium hypochlorite) before start of the testing and in between mice by 3% acetic acid to eliminate contaminating odors. Testing was conducted between 9 am and 6 pm daily.

#### 2.8.1. Open field test

Exploratory and anxiety-like behaviors were assessed over a 10 min session in an automated open field arena (40.6 cm × 40.6 cm; Kinder Scientific, Poway, CA). Interfaced infrared photobeams and Motor Monitor software allowed for calculation of parameters including total distance traveled and relative time spent in the more anxiety-provoking center versus the periphery of the open field (Semple et al., 2015a; Semple et al., 2015b). Testing was conducted between 9 am and 1 pm.

#### 2.8.2. Rotarod

The accelerating rotarod (Ugo Basile 7650, Gemonio VA, Italy) was used to assess motor function, coordination and motor learning, as previously described (Semple et al., 2015a, Semple et al., 2015b). Mice were placed on an accelerating rod that started at 5 rotations per minute. The latency to fall was recorded in *sec*, and mice were tested across three consecutive days, three trials per day with an inter-trial interval of approximately 1 h. Testing was conducted between 2 pm and 6 pm.

#### 2.8.3. Elevated plus maze

The elevated plus maze (Kinder Scientific) was used to assess anxiety-like behavior based on the natural tendency of rodents to avoid the open arms in preference for enclosed areas. Mice were placed individually in the center area of the maze and allowed free access for a 10 min period. Times spent in open versus closed arms was determined (Semple et al., 2015a, Semple et al., 2015b). Testing was conducted between 9 am and 1 pm.

#### 2.8.4. Resident-intruder test

To assess aggressive and defensive behaviors, a novel stimulus mouse (matched for strain, age and sex) was presented into the established home cage of the test mouse, and investigative behaviors were quantified from video recordings (Koolhaas et al., 2013). Testing was conducted between 2 pm and 6 pm.

#### 2.8.5. Morris water maze (MWM)

The MWM was used to assess spatial learning and memory as previously described (Pullela et al., 2006; Semple et al., 2015a; Semple et al., 2015b), using a 140 cm-diameter circular pool filled with opaque water (22 °C). Mice underwent two daily sessions (spaced 3.5 h apart – am sessions between 9 am and 1 pm and pm sessions between 2 pm and 6 pm) for 5 consecutive days. Each session consisted of three 60-s trials with a 10–15 min inter-trial interval. During days 1 and 2, the platform was raised above the water surface and clearly labeled with a flag (‘visible platform’). The platform location was rotated to a different quadrant in each session during this training stage, and mice that failed to reach the platform within 60 s were guided there by the investigator. During days 3, 4 and 5 (‘hidden platform’), the platform was hidden below the water surface and maintained in a constant location, such that mice were required to use spatial cues from the room to locate it. Movements were tracked with an overhead mounted video camera interfaced with Noldus EthoVision software (Noldus IT, Wageningen, The Netherlands), for quantification of the cumulative distance to the platform and latency to locate the platform, as well as swim speeds (cm/s). To assess spatial memory retention, at the conclusion of days 3, 4 and 5, ~ 1 h after completion of the last trial for that day, the platform was removed from the pool and a 60 s ‘probe trial’ was conducted. A fourth probe trial was performed 1 week later. As mice typically showed a preference for the target quadrant early in the probe trials and subsequently searched elsewhere, we analyzed only the first 30 s of each probe trial. Depending on the search patterns employed by the mice, time spent searching the target quadrant and cumulative distance to the target might differentially reflect memory retention in these probe trials; therefore, both performance measures were analyzed and presented. Finally, we analyzed thigmotaxis (time spent in the outer 20 cm zone of the pool), an indicator of anxiety-like behavior, during the visible platform and hidden platform trial and the probe trials.

### 2.9. Statistical analyses

Statistical analyses were performed using SPSS vs22 (Chicago, IL) or Prism v.8.0 (GraphPad Software, Inc., La Jolla, CA), with a significance level of *p* < 0.05. All data was analyzed for normality using Shapiro-Wilk test in GraphPad Prism before analysis and was found to be normally distributed. One- or 2-way analysis of variance (ANOVA) was used to compare 2 or more groups or factors (injury, genotype, and/or time), with stated post-hoc tests as appropriate. Repeated measures (RM) were used for the rotarod with factors of injury and session. Unpaired 2-tailed student’s *t*-tests were used for direct comparisons of 2 groups (between two genotypes or between injury and sham) as appropriate. Paired 2-tailed t-tests were used for direct comparisons between ipsilateral and contralateral hemispheres protein expression. The MWM learning curves were analyzed using a 2-way repeated-measures ANOVA with genotype and injury as between group variables. Performance during visible sessions and hidden platform sessions were analyzed separately. Cumulative distance to the target and latency to reach the target platform were used as performance measures. All statistical analyses for MWM were performed using SPSS vs22 software (Chicago, IL). *p*-Values of main effects from ANOVA’s are reported to 4 decimal points. *Post-hoc* analyses are stated as *p < 0.05, **p < 0.01, ***p < 0.001 ****p < 0.0001 and annotated graphically. Results are expressed as mean + standard error of the mean (sem).

#### 2.9.1. Factor analysis

As there was qualitative evidence that brain-injured animals may display thigmotaxis in the MWM, a principal components factor analysis (PCA) was performed to determine the relationships between performances on the various tasks on the level of individual mice, and was undertaken for two reasons: 1) to determine whether measures of activity or anxiety-like behavior, across injury conditions and genotypes, might contribute significantly to performance on learning and memory tasks, and 2) to approximate to what extent the distinct learning and memory task measures in the water maze measure the same underlying abilities in the mice. Measures entered into the model were: percentage time in the center of the open-field (anxiety measure), ratio times in the open arms of the elevated plus maze (anxiety measure), improvement in performance on the rotarod during training (motor learning measure), mean cumulative to the visible platform location in the water maze (task learning measure), mean cumulative distance to the hidden platform location in the water maze (spatial learning measure), percent time spent in the target quadrant in the first, second, third, and fourth probe trials (spatial memory retention measures), average percent time spent in the outer zone of the water maze during visible platform training (anxiety measure), average percent time spent in the outer zone of the water maze during hidden platform training (anxiety measure), average percent time spent in the outer zone of the water maze during the four probe trials (anxiety measure). The Principal components analysis was performed using SPSS, and factors with eigenvalue >1.0 were considered significant. The verimax rotated matrix was used to interpret the factor loadings.

## 3. Results

### 3.1. Prolonged recruitment of neutrophils is independent of Syk

**Fig. 1.**
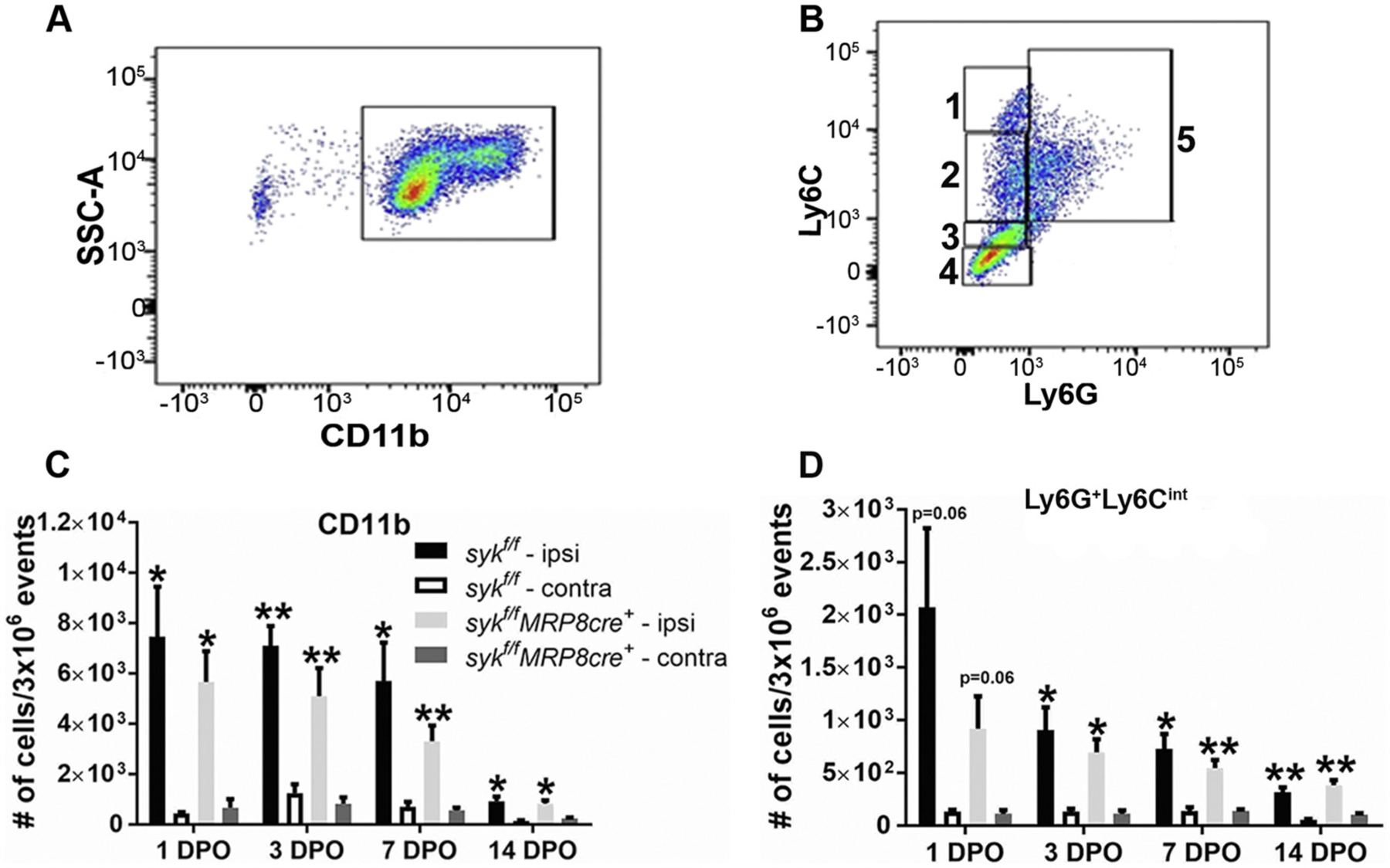
There is a prolonged recruitment of neutrophils into the brain over the first 14 days post-injury (DPO) that is independent of genotype. A, B). Gating was used to identify neutrophil subtypes (CD11b^+^, and Ly6G^+^ cells), 1. Ly6C^hi^Ly6G^−^ (pro-inflammatory monocytes); 2. Ly6C^int^Ly6G^−^ (anti-inflammatory monocytes); 3. Ly6C^lo^Ly6G^−^ (resident/patrolling macrophages); 4. CD11b^+^Ly6C^−^Ly6G^−^ (undifferentiated myeloid cells, microglia, dendritic cells); 5. Ly6G^+^ Ly6C^int^ (marker expressed on neutrophils and differentiating pre-monocytes) C–D). There are no genotypic differences in the temporal recruitment of CD11b+ cells (C) or neutrophils into the injured hemisphere [Ly6G^+^Ly6C^int^ (D)]. Bars represent mean + sem. Comparisons are between the ipsilateral and contralateral hemispheres (paired two-tailed *t*-test). As indicated on graph, *p < 0.05; **p < 0.01; ***p < 0.001; *n* = 5 mice/genotype/time-point. Comparisons between the ipsilateral hemispheres of the two genotypes (unpaired two-tailed t-test). All comparisons had *p* > 0.05.

We have previously shown by immunohistochemistry that injury to the developing brain results in prolonged recruitment of CD45+ and GR- 1+ cells into ipsilateral cortex within the first 14 days post-injury (Claus et al., 2010). In an effort to differentiate between the neutrophil and monocyte/macrophage populations, we used flow cytometry and the differential expression of Ly6C and Ly6G cell surface markers. Consistent with our previous data, injury at postnatal 21 (p21) resulted in a prolonged recruitment of CD11b+ leukocytes to the injured cortex (Fig. 1C). CD11b+ cells (Fig. 1A) were gated for differential expression of Ly6C (monocytes/macrophages) in the absence of Ly6G (Ly6C^−^, Ly6C^lo^, Ly6C^int^, and Ly6C^hi^) and also Ly6G+ neutrophils (Supplemental Fig. 2B and Fig. 1B and D, respectively).

To examine the role of Syk kinase in neutrophils, we compared responses in *syk*^*f/f*^ versus *syk*^*f/f*^*MRP8-cre*^+^ mice. There was sustained recruitment of CD11b+ cells in the ipsilateral as compared to the contralateral hemisphere for up to 14 days post-injury, with no statistically significant differences between genotypes at any of the time-points (Fig. 1C). Both myeloid cells (CD11b^+^Ly6C^−^Ly6G^−^) (Supplemental Fig. 2C) and neutrophils (Ly6G^+^Ly6C^int^) were likewise recruited into the injured brain over a similar period and showed no statistically significant differences between the genotypes at any of the time-points (Fig. 1B and D). While different populations of monocytes/macrophages were evident in the injured brains at variable times post-injury, there were no genotypic differences (Supplemental Fig. 2D–F). These data suggest that the prolonged recruitment of leukocytes into the injured brain is independent of Syk kinase expression in neutrophils.

We next determined if neutrophils, recruited in very high numbers at 24 h post-injury, expressed M2 markers (CD206, or arginase I). By flow cytometry, there was a low level of expression of CD206 on monocyte/macrophages and neutrophil subsets, whereas CD11b^+^Ly6C^−^Ly6G^−^ cell types expressed the highest level of CD206 (Supplemental Fig. 3A–C). Subsequent qualitative immunocytochemistry revealed absence of colocalization of arginase I in neutrophils (Ly6G^+^ cells; Supplemental Fig. 3D–F). By each of these independent measures, there were no differences between genotypes and our findings suggest that neutrophils, recruited into the injured brain at 24 h post-injury show modest to no expression of these classic anti-inflammatory markers.

### 3.2. Breakdown of the BBB is attenuated in Syk conditional knockouts

**Fig. 2.**
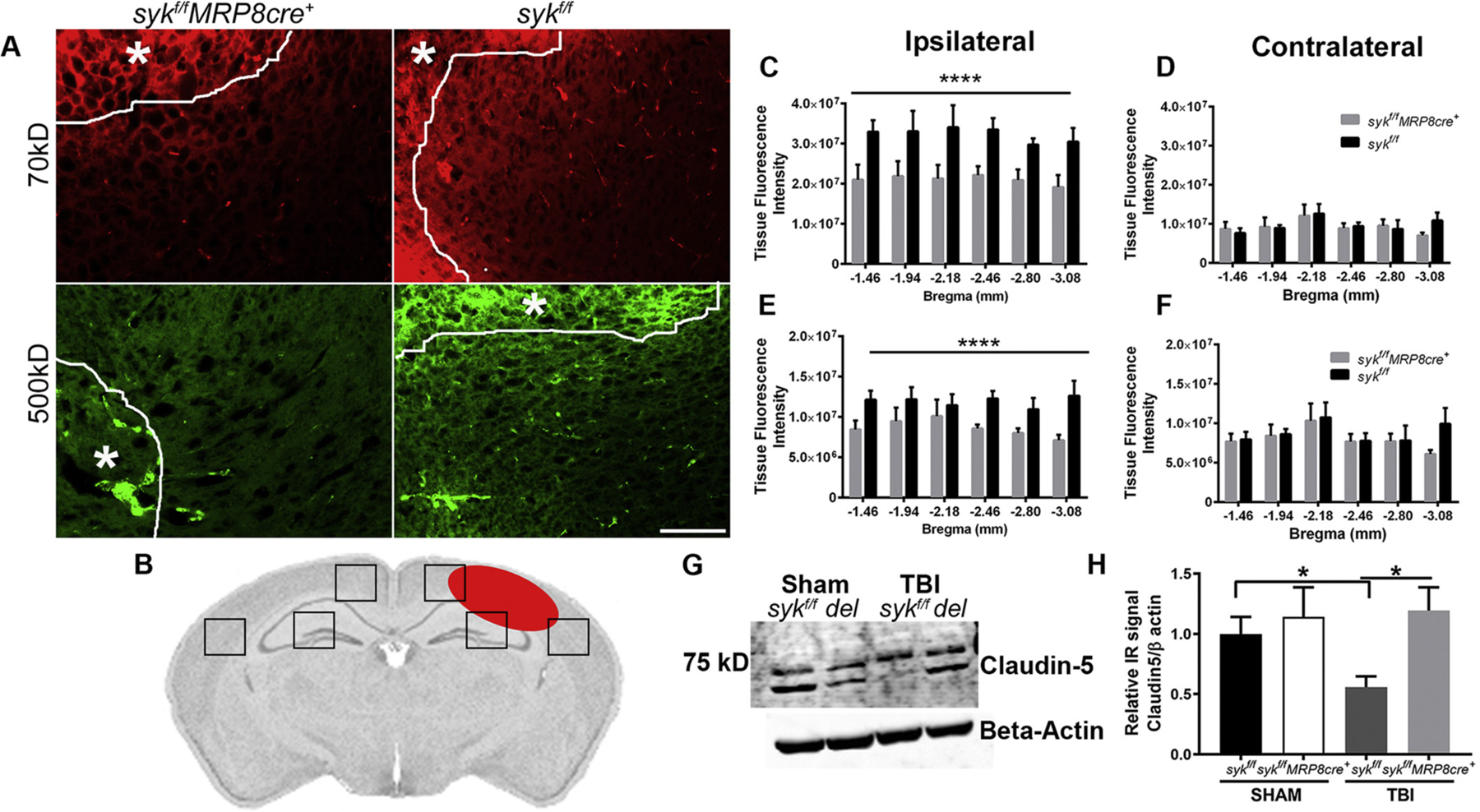
Disruption of the BBB to both low and high molecular weight dextrans is attenuated in the ipsilateral hemisphere of *syk*^*f/f*^*MRP8cre*^+^ mice at 24 h post-injury as compared to *syk*^*f/f*^ controls. A) Representative images of fluorescently tagged 70kD and 500kD dextrans at 1 day post-injury - in the ipsilateral hemisphere of *syk*^*f/f*^ and *syk*^*f/f*^*MRP8cre*^+^ mice. A white line and asterisk in each of the panels delineates the region that is closest to the site of injury. B) Schematic of a brain section that shows locations (boxes) in the ipsilateral and contralateral hemispheres that were used for quantification of fluorescence. The site of cortical injury is illustrated by a red oval structure. C) Tissue fluorescence intensity of 70kD dextran was significantly higher in *syk*^*f/f*^ as compared to *syk*^*f/f*^*MRP8cre*^+^ ipsilateral sections (Two way ANOVA, interaction n.s; effect of bregma n.s; effect of genotype ****p < 0.0001). D) There were no differences in fluorescence intensity of 70kD dextran between *syk*^*f/f*^ and *syk*^*f/f*^*MRP8cre*^+ is^ in sections that were contralateral to the injury (Two way ANOVA, n.s). E) Tissue fluorescence intensity of 500kD dextran was significantly higher in *syk*^*f/f*^ as compared to *syk*^*f/f*^*MRP8cre*^+^ ipsilateral sections (Two way ANOVA, interaction n.s; effect of bregma n.s; effect of genotype ****p < 0.0001). F) There was no difference in fluorescence intensity of 500kD dextran between *syk*^*f/f*^ and *syk*^*f/f*^*MRP8cre*^+^ in contralateral sections. *n* = 5 mice for *syk*^*f/f*^; *n* = 7 mice for *syk*^*f/f*^*MRP8cre*^+^. G, H) Claudin-5, a BBB tight junction protein, was quantified by Western immunoblots. A representative Western blot membrane at 24 h post-injury (G). Expression of claudin-5 at 24 h post-injury was notably reduced in the injured *syk*^*f/f*^ lysate as compared to its respective sham (two-tailed t-test, *p* = 0.0225) and injured *syk*^*f/f*^*MRP8cre*+ (two-tailed t-test, *p* = 0.0114) (H). Bars represent mean + sem; n = 5/group.

Neutrophils are recruited to the injured brain, peaking at 24 h post-injury (Claus et al., 2010), a time point that corresponds to a pronounced disruption of the BBB (Liu et al., 2018). It is thought that this abnormal permeability is due, at least in part, to the release of neutrophilic granular contents that degrade constituents of tight junctional complexes during paracellular transmigration (Price et al., 2016). Barrier disruption was quantitated in the cortex by fluorescence microscopy, using systemically administered fluorescent-tagged dextrans whose molecular weights of 70 kD and 500 kD approximated that of albumin and fibrinogen, respectively. While disruption of the barrier was apparent in both brain-injured genotypes, it was significantly attenuated in the *syk*^*f/f*^*MRP8cre*+ as compared to the injured *syk*^*f/f*^ mice (Fig. 2A–F; Supplemental Fig. 4). To determine if this reduction may be due to paracellular stabilization, we examined claudin-5, a key constituent of brain microvascular tight junctional complexes (Sweeney et al., 2019), by Western immunoblots of brain lysates. We chose to study claudin-5 as it plays a key role in the paracellular barrier to molecules. Claudin-5 is highly expressed in the brain (5× more as compared to other organs), and its loss, implicated in a variety of diseases, is due to the generation of reactive oxygen species and matrix metalloproteinases, each of which are generated by activated neutrophils (Greene et al., 2019). There was a significant preservation of the 62 kD band, representing activated dimers (Ishizaki et al., 2003), in the injured *syk*^*f/f*^*MRP8cre+* mice as compared to lysates from injured *syk*^*f/f*^ mice (Fig. 2G and H). These data suggest that loss of barrier integrity after TBI is, in part, attributed to Syk kinase-dependent degradation of inter-endothelial junctional proteins.

### 3.3. Syk conditional knockouts show decreased neutrophil ROS and spared NeuN protein levels

**Fig. 3.**
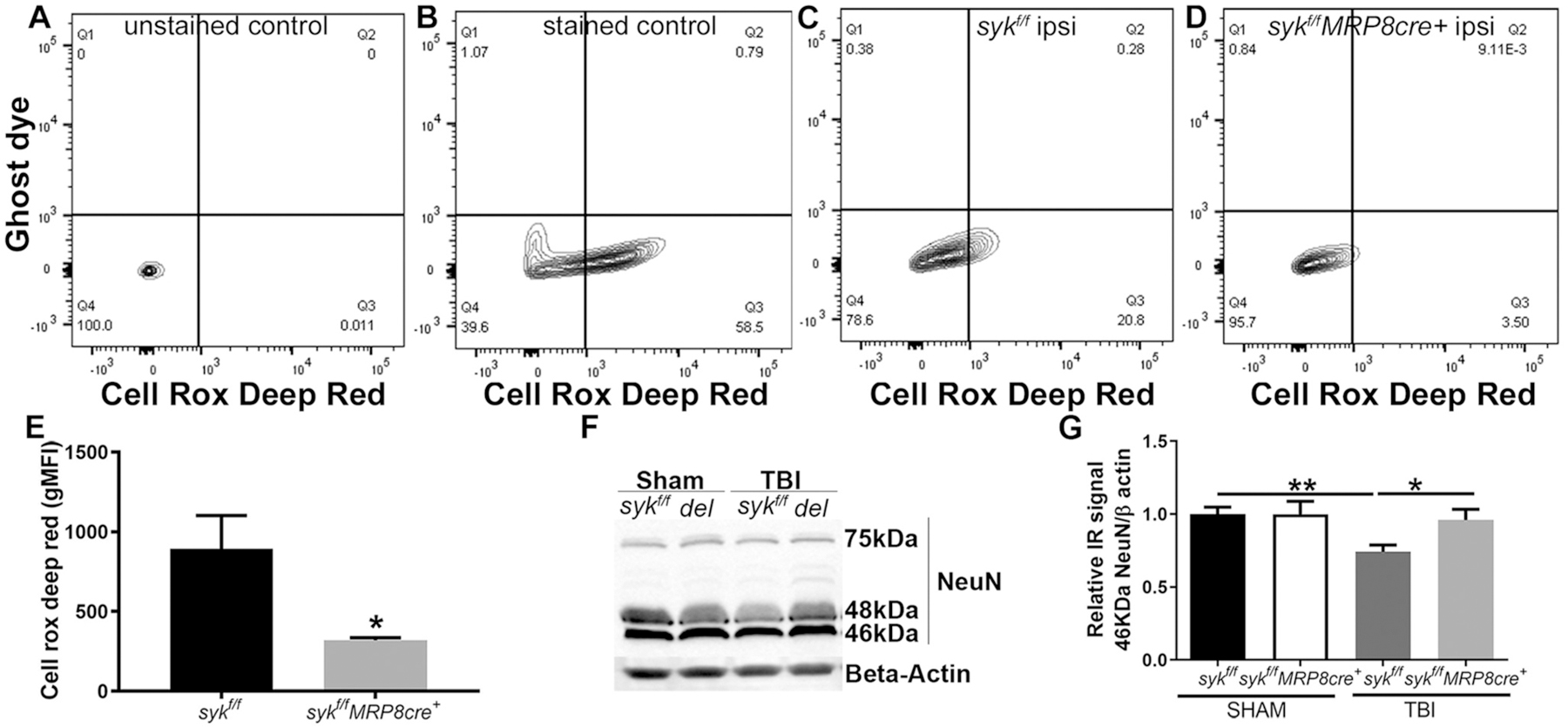
Neutrophil specific deletion of Syk confers acute neuroprotection and reduced generation of reactive oxygen species (ROS). Flow cytometry was used to detect ROS, using the CellROX^®^ Deep Red dye. This dye is non-fluorescent in a reduced state and exhibits bright fluorescence upon oxidation. A) The unstained brain lysate showed no fluorescence in the ROS detection channel. B) In the lysate, stained with ghost dye (live dead cell marker) and CellROX^®^ Deep Red dye, ROS positive signal was observed at 655 nm in ghost dye negative cells (live cells). C, D) Representative flow tracings indicate a more prominent shift in ROS positive signal in lysates from *syk*^*f/f*^ (C) as compared to *syk*^*f/f*^*MRP8cre*^+^ mice (D). E) Based upon the geometric mean fluorescence intensity, there was an elevated ROS signal in the *syk*^*f/f*^ as compared to *syk*^*f/f*^*MRP8cre*^+^ (unpaired two-tailed t-test, *p* = 0.0471) *n* = 6 mice/genotype. F, G) The neuronal marker NeuN was evaluated in the region adjacent to the site of impact by Western blot analysis at 24 h post-injury (F). The 46 kDa band of NeuN was reduced in the injured *syk*^*f/f*^ lysates as compared to injured *syk*^*f/f*^*MRP8cre*+ and compared to its sham (G) (two-tailed t-test, *p* = 0.0335 and *p* = 0.0032, respectively). Bars represent mean + sem; n = 5 mice/group. *p < 0.05; **p < 0.01.

As activated neutrophils release ROS which can contribute to secondary tissue damage, we quantitated neutrophil ROS in tissue digests by flow cytometry (Fig. 3A–E). Contralateral tissue punches spiked with blood were used as staining controls (Fig. 3A–B; without and with dye). Production of ROS in neutrophils was reduced in the injured *syk*^*f/f*^*MRP8cre*+ mice as compared to *syk*^*f/f*^ mice (Fig. 3C–E). To determine if reduced neutrophil ROS corresponded to neuronal survival, we evaluated the expression of NeuN, a marker of mature neurons, in the ipsilateral hippocampus in both genotypes and including sham and brain-injured mice. We focused on the hippocampus as we have previously shown that this structure shows a consistent pattern of neuronal injury/loss, likely independent of the primary insult, within the acutely injured young brain (Tsuru-Aoyagi et al., 2009). As previously reported, three distinct bands at 46, 48 and 75 kDa for NeuN were identified (Semple et al., 2015b). The 46 kDa band was quantified as it was the only band whose intensity was found to be injury-dependent in the hippocampal samples. Densitometric analyses revealed greater preservation of levels of the 46 kDa NeuN in the injured *syk*^*f/f*^*MRP8cre*+ mice as compared to the injured *syk*^*f/f*^ mice (Fig. 3F–G).

### 3.4. No genotypic differences in long-term volumetric loss of cortex and hippocampus

For long-term analyses, we first determined the overall health of mice as evidenced by weight gain over time. All animals gained weight and there were no differences in weight gain in response to injury or genotype (Supplemental Fig. 5). To determine whether absence of Syk kinase in neutrophils affects long-term loss of tissue after injury, the brains of *syk*^*f/f*^ and *syk*^*f/f*^*MRP8-cre*^+^ mice were examined at 2 months post-injury (~3 months of age) by stereological volumetric analysis. As might be expected, injury induced large volumetric losses in the ipsilateral dorsal cortex of both genotypes as compared to sham-operated mice. However, cortical volumes were similar in brain-injured *syk*^*f/f*^ and *syk*^*f/f*^*MRP8-cre*^+^ mice (Supplemental Fig. 6A). Volume of the contralateral dorsal cortex was also modestly reduced in response to injury (2-way ANOVA effect of injury F(1, 42) = 4.335, *p* = 0.0435) but was not affected by genotype (effect of genotype, F(1, 42) = 0.3054, *p* = 0.5834; injury x genotype interaction F(1, 42) = 1.145, *p* = 0.2907).

There was a similar injury-related reduction in volume of the ipsilateral dorsal hippocampus relative to respective shams in both genotypes (Supplemental Fig. 6B). Such a volumetric loss of the hippocampus was not apparent in the contralateral hemisphere in response to injury or genotype (2-way ANOVA injury x genotype interaction F(1, 42) = 0.01945, *p* = 0.8897; effect of injury F(1, 43) = 0.6766, *p* = 0.4153; effect of genotype, F(1, 43) = 0.9236, *p =* 0.3419). Subsequent measurements of the ipsilateral dorsal dentate gyrus (DG) likewise revealed a reduction in this structure in both genotypes, as compared to their respective shams (Supplemental Fig. 6C) with no differences in the contralateral DG by either injury or genotype (2-way ANOVA injury x genotype interaction F(1, 43) = 0.04264, *p* = 0.8374; effect of injury F (1, 43) = 0.03334, *p* = 0.8560; effect of genotype, F(1, 43) = 0.4207, *p* = 0.5200).

### 3.5. Long-term behavioral assessments

A series of behavioral tests were conducted at 2 months post-injury to assess long-term effects on sensorimotor function, overall activity, aggressive and defensive behaviors and cognitive function at adulthood.

#### 3.5.1. Syk conditional knockouts display improved motor learning

**Fig. 4.**
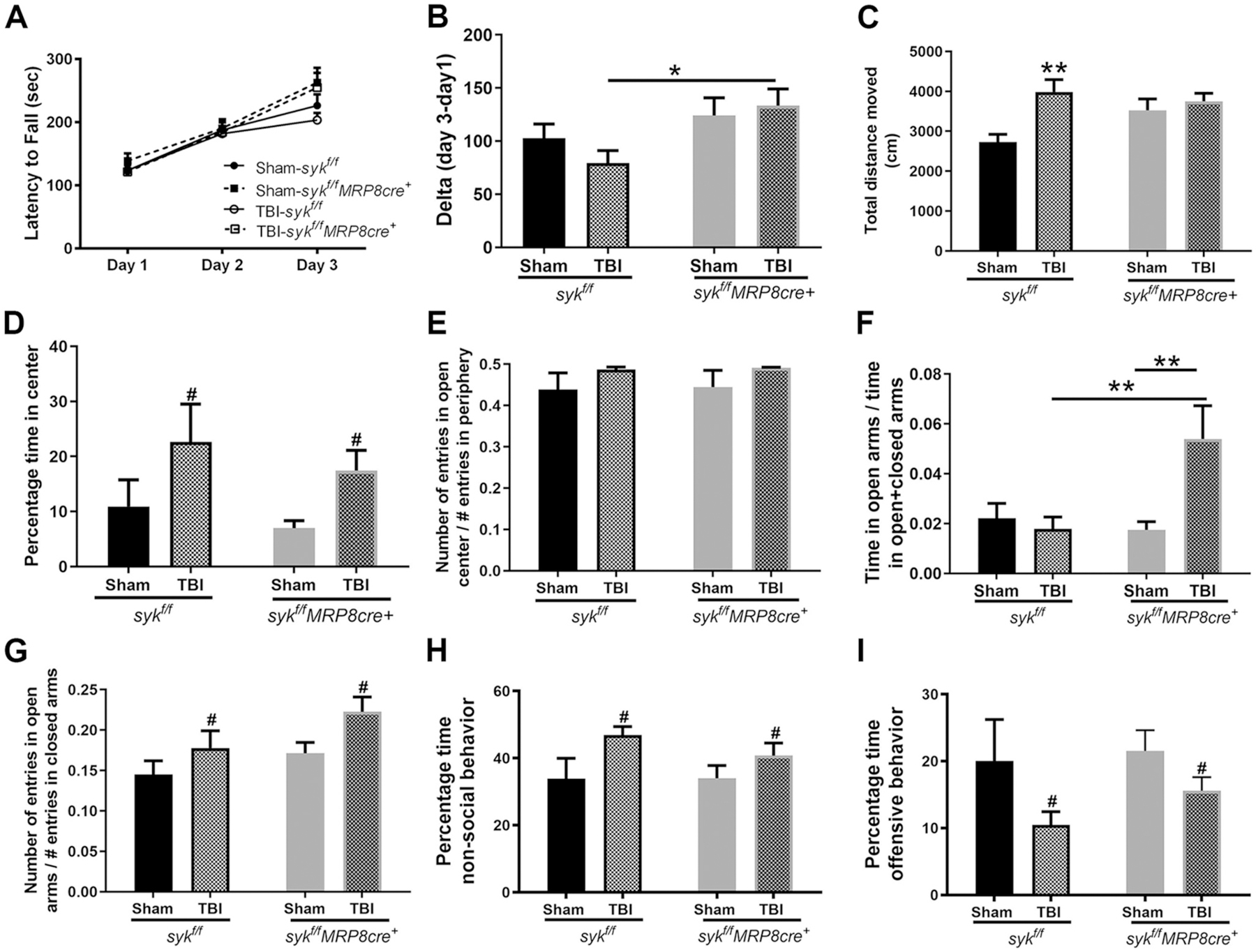
Injury induces long-term behavioral deficits at adulthood. A) Motor function (latency to fall), was evaluated based upon performance on a rotarod across 3 consecutive days. There was significant interaction, without an effect of injury or genotype, and all groups showed improvement over time (increased latency to fall) when evaluated across all time points (RM-2-way ANOVA, interaction *p* = 0.0201, effect of time, ****p < 0.0001, effect of injury/genotype *p* = 0.6135). B) However, in comparing 1 versus 3 days post-injury, injured *syk*^*f/f*^ mice showed significantly less improvement in rotarod performance with training than injured *syk*^*f/f*^*MRP8cre*^+^ mice (difference in time between day 3 and day 1) (2-way ANOVA: no significant interaction, effect of injury (ns), effect of genotype (*p* = 0.0148), followed by Sidak’s post-hoc test (*p = 0.0257 injured *syk*^*f/f*^ vs. injured *syk*^*f/f*^*MRP8cre*+ mice). C) There was an increase in distance traveled in the open field in brain-injured *syk*^*f/f*^ mice compared to sham controls (2-way ANOVA, interaction *p* = 0.0432; post-hoc analysis, Sidak’s test, **p = 0.0022). The percentage time spent in the center of open field was increased in brain-injured animals in both genotypes (2-way ANOVA, no significant interaction, effect of injury (*p = 0.0173), effect of genotype (ns)) (D). There were no significant effects on ratio entries in the center (2-way ANOVA, interaction = ns, effect of injury = ns, effect of genotype = ns) (E). In contrast, time spent in open arms of the plus maze revealed differences between injured *syk*^*f/f*^*MRP8cre*+ and sham control and injured *syk*^*f/f*^ mice (2-way ANOVA, interaction *p* = 0.0176; post-hoc analysis, Sidak’s test, *syk*^*f/f*^*MRP8cre*+ sham vs. injury **p = 0.0043, injured *syk*^*f/f*^ vs. injured *syk*^*f/f*^*MRP8cre*+ mice **p = 0.0059). (F) The ratio of entries in was increased in brain-injured animals in both genotypes (2-way ANOVA, no significant interaction, effect of injury (*p = 0.0221), effect of genotype (*p* = 0.0516)) (G). Social behavior was assessed by the resident intruder test. Brain-injured mice spent more time performing non-social behaviors as compared to sham controls, and this was comparable across both genotypes (2-way ANOVA, effect of injury *p = 0.0253) (H). Injured mice displayed submissive behavior when compared to shams and this was independent of genotype (I) (2-way ANOVA, effect of injury *p = 0.041) Values are mean + sem, *n* = 11–14/group.

Sensorimotor function was determined by performance on rotarod on three consecutive days at 2 months post-injury. All mice improved with time and there were no differences by injury or genotype (Fig. 4A). However, when we evaluated motor learning, as determined by change in latency to fall on day 3 compared to day 1, injured *syk*^*f/f*^*MRP8-cre*^+^ mice displayed longer latencies to fall as compared to injured *syk*^*f/f*^ mice (Fig. 4B), a finding supportive of improved motor learning.

#### 3.5.2. Injured mice display hyperactivity and reduced anxiety levels

These assessments were determined by evaluating performance in the open field and elevated plus maze. Hyperactivity and anxiety disorders are reported in children that have sustained TBIs (Chang et al., 2018; Max et al., 2011). Consistent with our previous findings (Semple et al., 2015b), the overall distance moved by brain-injured mice in an open field was greater than sham controls. However, these findings were limited to the injured *syk*^*f/f*^ mice as compared to their sham controls (Fig. 4C).

Anxiety-like behaviors were evaluated in the open field and the elevated plus maze. In the open field, thigmotaxis (time spent in the periphery) and reduced anxiety-like behavior (time spent in the center) were assessed. There was a similar reduction in anxiety-like behavior across both injured groups as compared to genotype-matched shams (Fig. 4D). Since, the overall activity levels were altered in response to injury, we then determined if this was reflected in the number of entries to the center as compared to the periphery of the open field. There were no injury or genotype-induced differences in this measure (Fig. 4E). In a complimentary approach, we assessed the ratio of time spent in open arms of a plus maze over the time spent in the open and closed arms. Reduced anxiety-like behavior (preference for the open arms) was only apparent in brain-injured *syk*^*f/f*^*MRP8-cre*^+^ mice, as this ratio was higher as compared to their sham controls and injured *syk*^*f/f*^ mice (Fig. 4F). When we compared the ratio of entries (number of entries into the open arms divided by the entries in the open and closed arms), the ratio was higher in injured mice than sham mice, with no difference between genotypes (Fig. 4G). Collectively, these findings demonstrate that an early age TBI results in reduced anxiety-like behavior in the open field that is independent of genotype. However, testing in the elevated plus maze revealed genotypic differences; reduced anxiety-like behavior was evident in brain-injured *syk*^*f/f*^*MRP8-cre*^+^ mice compared to injured *syk*^*f/f*^ mice.

#### 3.5.3. Injury induced non-social behavior is genotype independent

The resident intruder paradigm was used to measure aggressive and defensive-like behaviors (Koolhaas et al., 2013). Adult male rodents display a natural offensive aggressive-like behavior to establish territory, social dominance and protect their resources (Koolhaas et al., 2013). Brain-injured mice, independent of genotype, spent more time performing non-social behaviors (such as self-grooming, and exploring the cage away from intruder) (Fig. 4H) and submissive-like behaviors (as defined by retreating in response to an attack by the intruder) compared to their sham controls (Fig. 4I).

#### 3.5.4. Spatial learning and memory deficits are rescued in Syk conditional knockouts

**Fig. 5.**
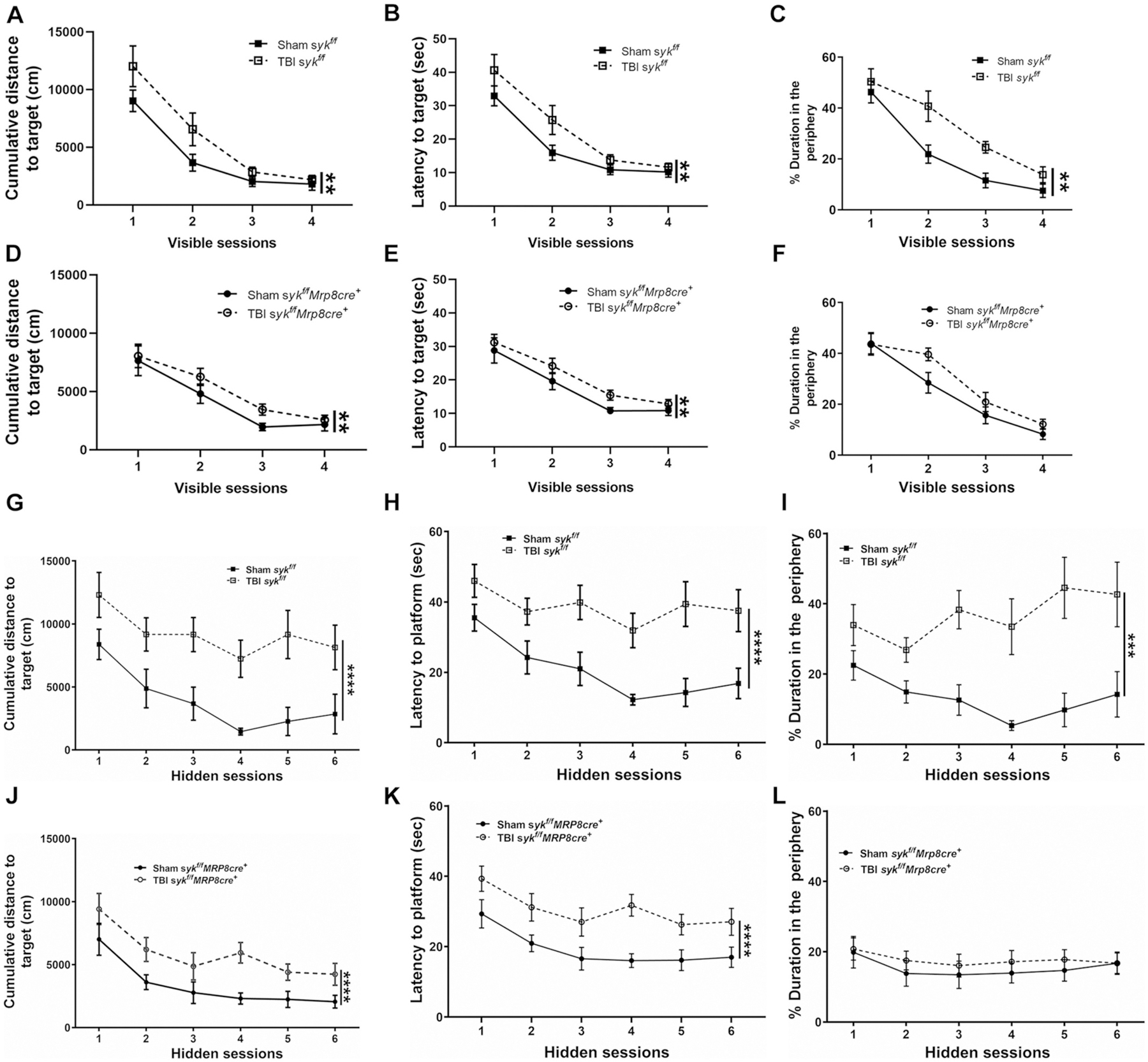
Injury induced changes in long-term deficits in spatial learning, working memory and short and long-term memory retention. The Morris water maze (MWM) was performed to assess spatial learning and memory. In this task, all mice showed improvement over time during the visible platform sessions, although brain-injured mice swam longer distances (RM two way ANOVA, effect of injury **p = 0.008) (A & D) and exhibited longer latencies to reach the platform overall (B & E) (RM two way ANOVA, effect of injury **p = 0.004) compared to sham controls, indicating impaired task learning after injury. During visible platform trials there was increased thigmotaxis in brain-injured mice as compared to their sham controls (RM two way ANOVA, effect of TBI (*F* = 5.154, *p* = 0.010) (C & F). When the platform was hidden in subsequent sessions, brain-injured mice again exhibited longer cumulative distances to reach the platform (RM two way ANOVA, effect of injury ****p < 0.0001) (G & J) and a longer latency to reach the platform (H & K) (RM two way ANOVA, effect of injury ****p < 0.0001) as compared to sham controls, indicating poorer spatial memory. During hidden platform trials, only *syk*^*f/f*^ injured mice displayed greater thigmotaxis as compared to shams (*F* = 9.910, *p* = 0.01) (I & L). n = 11–13/group.

**Fig. 6.**
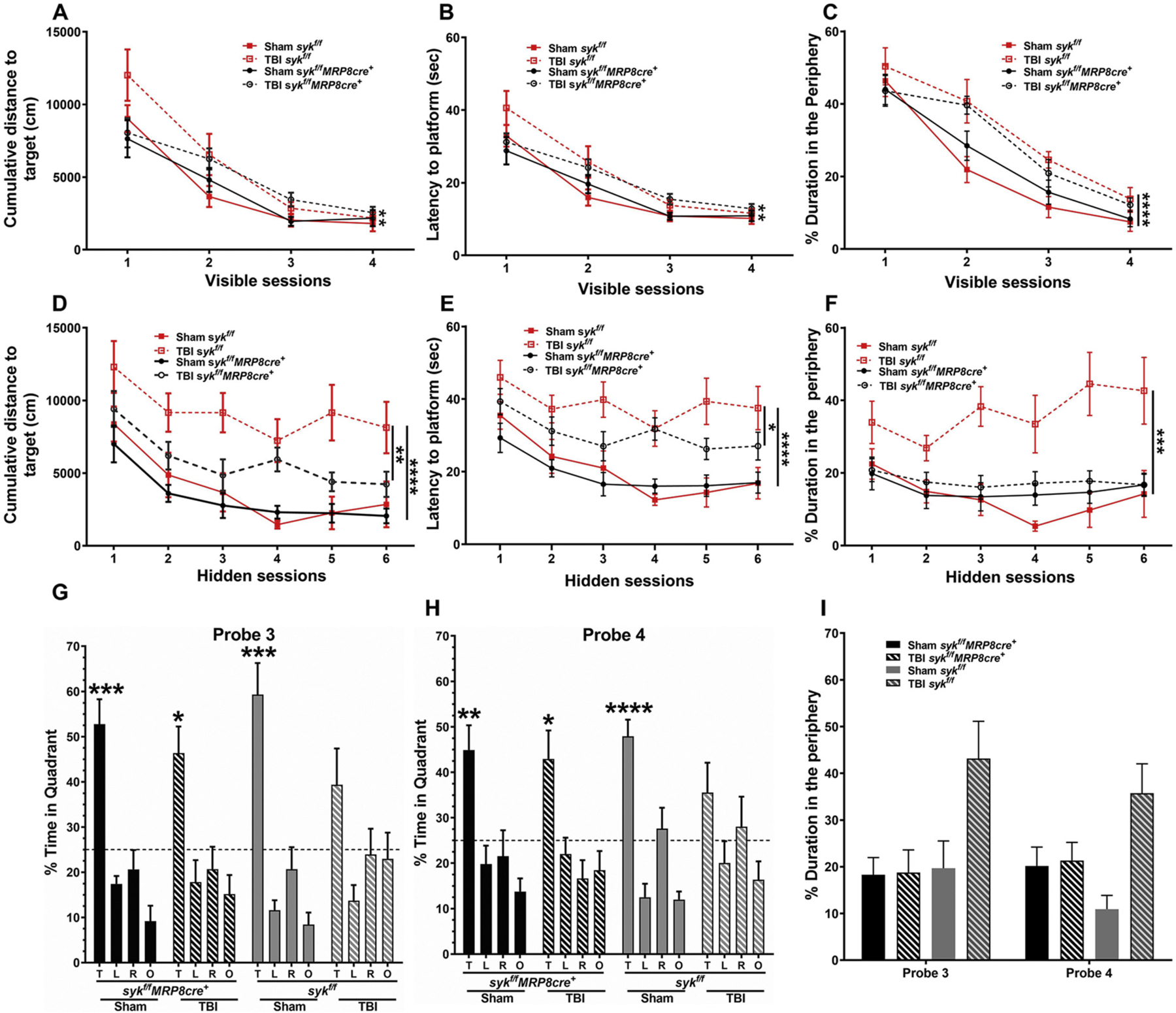
TBI induced long-term deficits in spatial learning, working memory and short and long-term memory retention, are rescued in *syk*^*f/f*^*MRP8cre*^+^ mice. The Morris water maze (MWM) was performed to assess spatial learning and memory. In this task, all mice showed improvement over time during the visible platform sessions, although brain-injured mice swam longer distances (RM two way ANOVA, effect of injury **p = 0.008) (A) and exhibited longer latencies to reach the platform overall (B) (RM two way ANOVA, effect of injury **p = 0.004) compared to sham controls, indicating impaired task learning after injury. During visible platform trials there was increased thigmotaxis in brain-injured mice as compared to their sham controls (RM two way ANOVA, effect of TBI (*F* = 5.154, *p* = 0.010). (C) When the platform was hidden in subsequent sessions, brain-injured mice again exhibited longer cumulative distances to reach the platform (RM two way ANOVA, effect of injury ****p < 0.0001) (D) and a longer latency to reach the platform (E) (RM two way ANOVA, effect of injury ****p < 0.0001) as compared to sham controls, indicating poorer spatial memory. Furthermore, injured *syk*^*f/f*^*MRP8cre*+ mice swam significantly shorter distances to the platform (RM two way ANOVA, effect of genotype **p = 0.004) and had shorter latencies to reach the platform (RM two way ANOVA, effect of genotype *p = 0.037) as compared to the injured *syk*^*f/f*^ mice. During hidden platform trials, only *syk*^*f/f*^ injured mice displayed greater thigmotaxis as compared to shams as well as injured *syk*^*f/f*^*MRP8cre*+ mice. There was an effect of TBI (*F* = 8.871, *p* = 0.001) and a genotype x TBI interaction (*F* = 7.241, *p* = 0.002). Therefore, we also assessed the effects of TBI on thigmotaxis in each genotype separately. There was an effect of TBI in *syk*^*f/f*^ (*F* = 9.910, *p* = 0.01) with greater thigmotaxis in TBI than sham mice but not in *syk*^*f/f*^*MRP8-cre*^+^mice (*F* = 1.187, *p* = 0.323) (F) Sham and brain-injured mice were tested for memory retention in Probe trial 3. Similar to shams of both genotypes, brain-injured *syk*^*f/f*^*MRP8cre*+ mice spent more time in the target quadrant (T) as compared to other quadrants, whereas brain-injured *syk*^*f/f*^ mice showed no preference between quadrants (RM one way ANOVA with Dunnett’s test, statistics as shown on graph *p < 0.05, ***p < 0.001) (G). K) Probe trial 4 was conducted 1 week after Probe trial 3. Injured *syk*^*f/f*^*MRP8cre*+ mice spent more time in the target quadrant compared to other quadrants, similar to both sham groups, whereas, brain-injured *syk*^*f/f*^ mice showed no preference (RM one way ANOVA with Dunnett’s test, statistics as shown on graph *p < 0.05, **p < 0.01, ****p < 0.0001) (H). When thigmotaxis was analyzed during the probe trials, there was an effect of TBI (*F* = 4.451, *p* = 0.018) and a genotype x TBI interaction (*F* = 10.064, *p* < 0.001). There was an effect of TBI in *syk*^*f/f*^ (*F* = 12.740, *p* < 0.001) with greater thigmotaxis in TBI than sham mice but not in *syk*^*f/f*^*MRP8-cre*^+^mice (*F* = 0.743, *p* = 0.487) (I). n = 11–13/group.

We next determined if activated neutrophils, recruited into the acutely injured brain, have long-term consequences on spatial learning and memory in the MWM. There were no injury or genotype-dependent differences in swim velocity (Supplemental Fig. 7A). The ability of mice to learn was first evaluated in visible platform trials by analyzing cumulative distance to the target and latency to the target. Brain-injured mice showed poorer learning curves as compared to shams, with no differences between genotypes. There were longer latencies and increased cumulative distances to reach the visible platform over trials 1–3 in brain-injured animals, as compared to sham controls (Fig. 5A, B, D–E). However, by visible platform training trial 4, all injured mice performed similar to shams. Furthermore, there were no differences between both the injured genotypes (Fig. 6A–B). We assessed the path traveled by measuring thigmotaxis across all groups during the visible platform trials. Thigmotaxis was greater in brain-injured mice compared to their sham controls with no effect of genotype (Fig. 5C). Overall, injured mice showed genotype independent deficits in task learning and increased thigmotaxis, both of which improved over time (Figs. 5A–F and 6A–C).

The hidden platform sessions were used to assess acquisition (spatial learning) by analyzing two independent performance measures, cumulative distance and latency to reach the target platform; sham mice showed better performance, based upon these metrics, than brain-injured mice (Fig. 5G, H, J, K). Furthermore, injured *syk*^*f/f*^*MRP8-cre*^+^ mice had shorter cumulative distance and latencies to find the target hidden platform than injured *syk*^*f/f*^ mice (Fig. 6D & E). When thigmotaxis was analyzed during hidden platform training, a similar pattern was observed. In injured *syk*^*f/f*^*MRP8-cre*^+^mice, thigmotaxis was similar to its sham control (Fig. 5L). In contrast, injured *syk*^*f/f*^ mice had increased thigmotaxis as compared to its sham control (Fig. 5I). Importantly, brain-injured *syk*^*f/f*^*MRP8-cre*^+^mice showed improved performance on hidden platform and decreased thigmotaxis as compared to brain-injured *syk*^*f/f*^ mice, suggesting a rescue of spatial learning deficits and anxiety-like behavior (Fig. 6D–F).

Four probe trials were next conducted to assess spatial memory, as determined by time spent in the target quadrant relative to the other quadrants. In probe trials 1 and 2, performed at the end of hidden trial days 1 and 2, only sham mice for each of the genotypes showed preference for the target quadrant; a strong trend (*p* = 0.0501) was observed in probe trial 2 for brain-injured *syk*^*f/f*^*MRP8-cre*^+^ mice (Supplemental Fig. 7B, C). In probe trial 3, sham mice showed preference for the target quadrant. Furthermore, only brain-injured *syk*^*f/f*^*MRP8-cre*^+^ mice showed a similar preference for the target quadrant (Fig. 6G). Without any further training, probe trial 4 was conducted a week later to assess long-term memory retention. The findings were similar to those in probe trial 3, with sham controls, independent of genotype, and brain-injured *syk*^*f/f*^*MRP8-cre*^+^ mice showing preference for the target quadrant (Fig. 6H). Thigmotaxis, analyzed during the probe trials, was greater in brain-injured *syk*^*f/f*^ mice compared to sham controls. However, a similar distinction was not evident in injured *syk*^*f/f*^*MRP8-cre*^+^mice compared to their controls (Supplemental Fig. 7D and Fig. 6I). These data, together with the analyses of thigmotaxis during probe trials, further confirm a rescue of the detrimental effects of TBI on spatial memory in *syk*^*f/f*^*MRP8-cre*^+^mice.

### 3.6. Relationships between behavioral and cognitive measures on the various tasks

Given the data regarding thigmotaxis during probe trials (described above), a principle components analysis was performed to determine whether measures of activity or anxiety-like behaviors across injury conditions and genotypes might have contributed to performance in the MWM test and to approximate to what extent the distinct performance measures in the MWM reflect similar underlying abilities in the mice.

This analysis revealed five factors with eigenvalues >1.0, which explained a total of 74.4% of the variance among the measures entered into the model (Table 1). The five factors explained 20.9, 15.0, 14.7, 14.5, and 9.4% of the variance, respectively. Percent time spent in the center of the open field and mean cumulative distance in the water maze during hidden platform training, average percentage time spent in the outer zone of the water maze during hidden platform training and probe trials all loaded onto Factor 1, indicating a common underlying ability being measured by all of these tests. The directions of the component loadings in Factor 1 were such that increasing values of the factor increased time spent in the center of the open field and decreased measures of cognitive performance. It is important to realize that in the open field, the mouse is put in the center of the open field at the beginning of the test. Therefore, more time spent in the center of the open field could reflect reduced activity levels as well as reduced anxiety measures. In contrast, in the elevated plus maze, the mouse is put in the center of the plus maze, an area that is not considered open or closed and not taken into consideration for calculating ratio times. Therefore, the ratio times in the elevated plus maze could reflect more measures of anxiety rather than activity as compared to the center time measure in the open field. Ratio times in the arms of arms of the elevated plus maze and percent time in the target quadrant in the third and fourth probe trials loaded on Factor 2. The directions of the component loadings in Factor 2 were such that reduced measures of anxiety were associated with increased measures of cognitive performance. The fact that percent time spent in the center of the open field and ratio times in the elevated plus maze loaded on different factors indicate that these two behavioral tests measure different underlying attributes in the mice. As described above, some of these differences might relate to where in the maze the mouse is put at the beginning of the test. Cumulative distance to the visible platform in the water maze and percent time spent in the outer zone of the water maze during visible platform training in the water maze loaded on Factor 3. The directions of the component loadings in Factor 3 were such that in the MWM increased measures of anxiety were associated with decreased measures of cognitive performance. Factors 1–3 indicate that there might be an anxiety-related component to performance in the water maze. Cumulative distance to the hidden platform in the water maze and percent time spent in the target quadrant in the first and second probe trials loaded on Factor 4. The directions of the component loadings in Factor 4 were such that improved spatial learning was associated with improved spatial memory retention. Finally, total distance moved in the open field and the improvement in rotarod performance loaded on Factor 5. The directions of the component loadings in Factor 5 were such that higher activity levels were associated with improved performance with training on the rotarod.

## 4. Discussion

These studies reveal a genotype independent, sustained recruitment of distinct leukocyte subsets including Ly6G^+^/Ly6C^int^ after traumatic injury to the developing brain. While the magnitude of recruitment is similar between genotypes, BBB disruption, generation of ROS and neuronal damage/loss are significantly reduced in the acutely injured brains of *syk*^*f/f*^*MRP8-cre*^+^ mice as compared to *syk*^*f/f*^ mice. At adulthood, brain-injured *syk*^*f/f*^*MRP8-cre*^+^ mice show robust improvements in hippocampal-dependent acquisition and short and long-term retention in spatial memory. These data establish for the first time a mechanistic link between neutrophil activation, acute pathogenesis, and long-term functional recovery after traumatic injury to the developing brain.

### 4.1. Neutrophils and BBB disruption

Neutrophil recruitment and neutrophil-secreted proteases are involved in disease progression after traumatic injury to the developing brain (Semple et al., 2015a; Semple et al., 2015b). Neutrophils are rarely found in the parenchyma of the normal brain with very low numbers in the cerebrospinal fluid and within the meninges (Liu et al., 2018). After TBI, neutrophils may gain access to the brain as a result migration from the meninges as well as transendothelial or paraendothelial pathways (Louveau et al., 2015).

Neutrophils are recruited to the acutely injured brain, coinciding with the time course of disruption of the BBB to circulating proteins and an increase in brain water content, the classic features of vasogenic edema (Michinaga and Koyama, 2015). However, whether or not neutrophils are directly responsible for vasogenic edema remains unclear. Early studies, utilizing various immunodepletion and genetic strategies to examine the contribution of neutrophils to BBB disruption after TBI have yielded varying results. Brain-injured mice, genetic deficient in dual P-selectin/ICAM-1, showed no change in the accumulation of neutrophils in the injured brain but did result in a reduction in vasogenic edema, as evidenced by a decrease in water content (Whalen et al., 1999). While immunodepletion of neutrophils with the Ly6G/C (antiGR-1) antibody also resulted in a reduction in water content, the barrier remained disrupted to the intravascular tracer Evan’s blue-bound albumin (EBA) (Kenne et al., 2012). Similar findings of reduced brain water content, despite abnormal permeability to EBA were reported in brain-injured adult CXCR2 knockouts (Semple et al., 2010) that lack the main receptor in regulating neutrophil chemotaxis (Li et al., 2015). While these early studies have implicated neutrophils in vasogenic edema, several issues remain. It is unclear why barrier disruption to a circulating protein remained unchanged, despite a reduction in brain water content. Additionally, these models either lacked specificity to neutrophils or may have been subject to compensatory mechanisms. The Ly6G/C antibody depletes neutrophils, activated monocytes and some macrophages (Daley et al., 2008). Mice deficient in CXCR2 may show compensatory mechanisms which could contribute to these disparate outcomes. For example, CXCR2 deficient mice show a pronounced inflammatory response to both cutaneous and peritoneal inflammatory stimuli, resulting from a reduced expression of cytokines during the resolution phase of inflammation as well as an increase in accumulation of macrophages at the sites of inflammation (Dyer et al., 2017). Here we have pursued an alternative strategy to better understand the contributions of neutrophils to barrier disruption after an early age TBI using mice with a conditional deletion of *syk* that is unique to neutrophils and which renders them unable to assume an activation state. We find that abrogation of Syk kinase expression stabilizes the BBB after injury but does not alter the magnitude of recruitment of neutrophils or the monocyte/macrophage population to the injured brain up to 14 days post-injury. Such findings suggest Syk kinase independent pathways including transendothelial entry and/or migration from the meningeal compartment.

### 4.2. Neutrophils as targets for stabilizing the BBB and reducing ROS

Cerebrovascular dysfunction, including disruption of the BBB to circulating proteins and development of vasogenic edema, is a hallmark of secondary pathogenesis in brain-injured children (Jullienne et al., 2016; Pop and Badaut, 2011). In earlier studies, we found that neutrophil elastase, a potent protease expressed and stored in neutrophil granules and released upon activation of these leukocytes, contributes to vasogenic edema after an early age TBI (Semple et al., 2015b). Building upon these findings, we now show that the *syk* conditional knockout exhibits a TBI-dependent reduction in barrier disruption to albumin- and fibrinogen-sized dextrans at 24 h post-injury. Interestingly, this stabilization of the barrier also coincides from greater preservation of the endothelial tight junction protein claudin-5, which has been linked with peak disruption of the BBB after an early age TBI (Badaut et al., 2015). The innate immune response and neutrophils, in particular, play an important role in development of secondary pathogenesis (Liu et al., 2018). In the context of the BBB, neutrophil activation and the release of granular content may be the driving force for its disruption after TBI. Release of azurocidin, a microbicidal peptide, from secretory vesicles and azurophilic granules, containing defensins and serine proteases, increase vascular permeability by a direct action on endothelial cells (Di Gennaro et al., 2009; Soehnlein and Lindbom, 2009). Degradation of the extracellular matrix proteins, tight junction proteins such as claudins, and adherens junction such as VE cadherin, occurs by proteases released from granules (upon neutrophil activation) that in turn results in endothelial barrier disruption (Kurz et al., 2016; Wang et al., 2005; Young et al., 2007). Our findings of greater preservation of claudin-5 in the conditional knockout, coincident with reduced barrier disruption to circulating proteins, are consistent with the ability of activated neutrophils to disrupt the barrier through degradation of endothelial tight junctions.

TBI results in an early increase in generation of ROS that likely causes further damage to the surrounding tissue (Abdul-Muneer et al., 2015). Resident microglia and infiltrating neutrophils and macrophages are a major source of ROS after TBI (Liu et al., 2018). The developing brain is particularly vulnerable to oxidative stress (Ditelberg et al., 1996; McLean et al., 2005; Mischel et al., 1997; Tsuru-Aoyagi et al., 2009). This increased susceptibility of the developing brain may be due to several key events; namely, excessive pro-oxidant potential given the presence of free iron and polyunsaturated fatty acids (Coyle et al., 1993), which in turn produce reactive hydroxyl radicals and ROS and lipid peroxidation (Potts et al., 2006; Siddappa et al., 2002), and developmentally regulated, inadequate antioxidant reserves (Tsuru-Aoyagi et al., 2009). Elevated ROS have broad effects on the brain including altered cerebral blood flow and brain oxygenation and disruption of the BBB, neuronal loss, and neuroinflammation (Nasr et al., 2019). Activation of neutrophils is dependent on Syk signaling and results in release of specific granules for production of ROS (Lowell, 2011). Here we show that neutrophils lacking Syk show reduced generation of ROS with greater preservation of NeuN by Western immunoblots in the acutely injured brain as well as with long-term improvements in learning and memory.

### 4.3. The Syk kinase signaling pathway: activation rather than altered recruitment

**Fig. 7.**
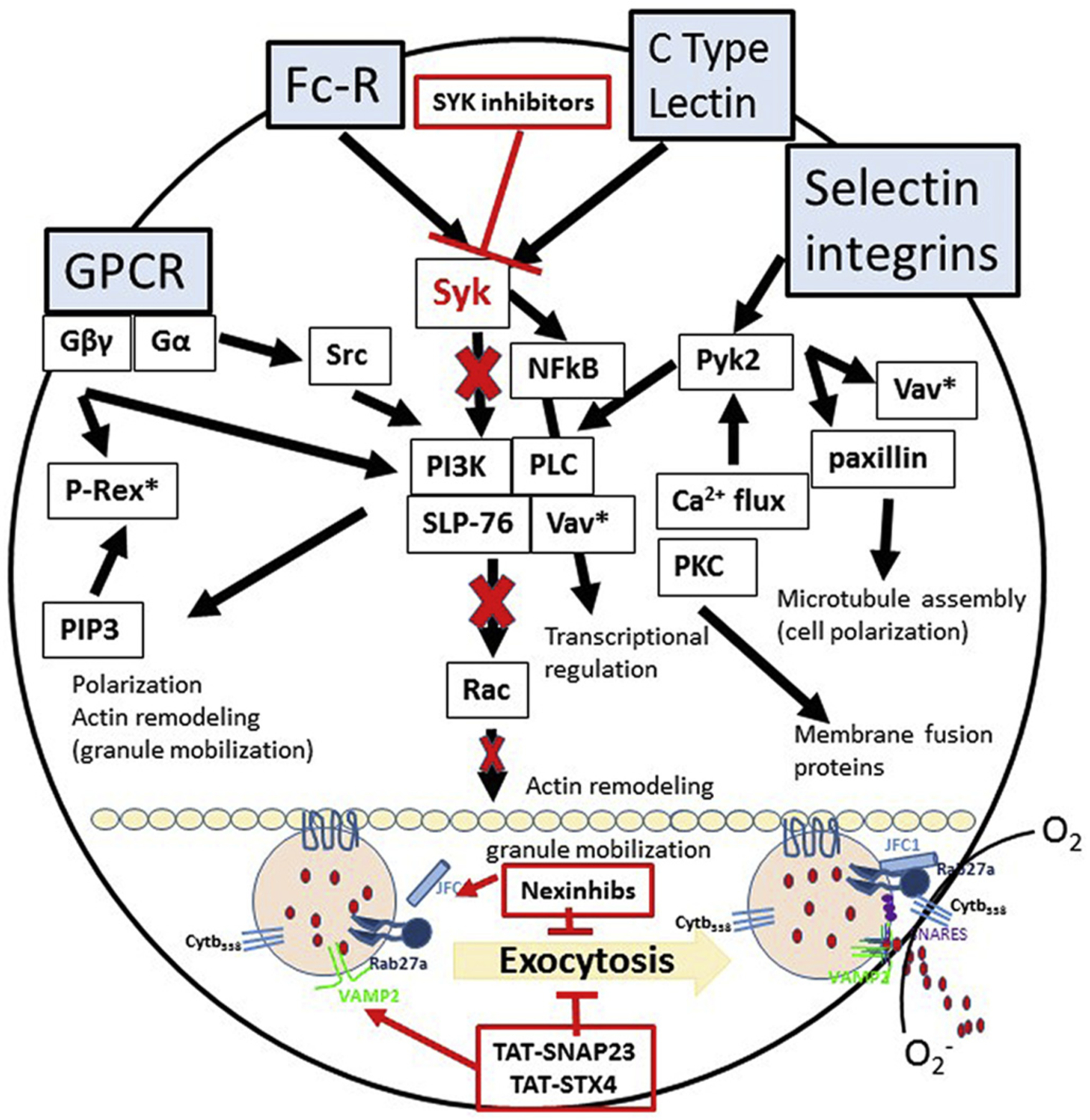
Diagrammatic representation of neutrophil activation and degranulation associated signaling via Syk pathway. Activation of neutrophils occurs through the binding of ligands to various cell surface receptors (Fc-R, integrins, and C-type lectins). GPCR activation triggers phospholipase Cβ (PLCβ)-mediated Ca2+ signals and activates phosphoinositide 3-kinase (PI3K). Fcγ and Fcα receptors signal through immunoreceptor tyrosine-based activation motif (ITAM)-mediated activation of the tyrosine kinase SYK; whereas, selectin and β2 integrin based ligand binding triggers tyrosine kinase pathways through SRC and SYK family kinases. Phosphorylation of SYK results in activation of the Rac signaling pathway and actin cytoskeleton reorganization and microtubule polarization. This triggers the mobilization of granules for the final steps in degranulation. Granule secretion involves the interaction of Rab27a with JFC1 and v-SNAREs. In the Syk KO mice, as used in this study; much of the downstream signaling is diminished (See X’s in red), resulting in a reduction of degranulation. Nexinhib, neutrophil-specific exocytosis inhibitor; SNARE, soluble *N*-ethylmaleimide-sensitive factor attachment protein receptor; TAT-SNAP-23, fusion proteins containing cell penetrating peptide of HIV TAT and the N-terminal SNAP-23 SNARE domain; TAT-STX-4, fusion proteins containing cell penetrating peptide of HIV TAT and the syntaxin-4 SNARE domain; VAMP, vesicle-associated membrane protein. This figure was adapted from Sheshachalam, A, et al., 2014 and Catz, SD and McLeish, KR 2020).

In an effort to address the molecular mechanisms involved in neutrophil-directed pathogenesis, we focused on Syk kinase, which is central to intracellular signaling for neutrophil activation downstream of a number of activating receptors (Fig. 7). Signaling events triggered by integrin receptors depend on Syk function to induce degranulation and ROS production (Catz and McLeish, 2020; Mocsai et al., 2002). A logical next step is to use a Syk inhibitor to further explore the interactions of neutrophils with other cells in the injured brain. Here we report that neutrophils show a prolonged recruitment into the injured young brain, spanning both the acute phase as well as early wound healing. While we demonstrate that these leukocytes are damaging to the acutely injured brain, their involvement in wound healing remains unclear. Thus, temporally defined inhibition of Syk, will elucidate any potentially divergent roles in acute injury versus the wound healing phase. In this study we found no differences in the temporal recruitment of neutrophils between *syk*^*f/f*^ versus *syk*^*f/f*^*MRP8-cre*^+^ mice by flow cytometry. Consistent with these data, *syk*^−*/*−^ neutrophils have been reported to undergo normal migration in several in vitro assays and disease models (Elliott et al., 2011). Moreover, the *syk*^*f/f*^*MRP8-cre*^+^ mice have been shown to exhibit normal levels of circulating monocytes and neutrophil recruitment following an inflammatory stimulus but display reduced activation of neutrophils(Elliott et al., 2011; Van Ziffle and Lowell, 2009). Hence, the protective effect observed acutely and long-term after injury to the developing brain in *syk*^*f/f*^*MRP8-cre*^+^ mice is likely a result of dampened activation of neutrophils rather than a migratory deficit. This is supported by the observation that the injured cortical tissue from *syk*^*f/f*^*MRP8-cre*^+^ mice showed reduced production of ROS and improved BBB function including claudin 5 expression as compared to *syk*^*f/f*^ mice.

### 4.4. Neutrophil phenotype in the acutely injured brain

We have previously shown that neutrophils, identified by immunolocalization of GR-1 and their classic lobulated nuclei, are recruited to the injured brain over the first several weeks post-injury (Claus et al., 2010). The present study, employing flow cytometry, validated these earlier findings. Additionally, we examined the phenotype of these leukocytes, based upon studies of tumor associated neutrophils (TANs) that have revealed their functional plasticity. Similar to macrophages, TANs express either a pro-tumoral (anti-inflammatory, N2) or anti-tumoral (pro-inflammatory, N1) phenotype (Mantovani, 2009; Nemeth et al., 2020). The mannose receptor, CD206, is differentially expressed on immune cells, depending on their polarization. Low levels of expression suggest a pro-inflammatory phenotype, whereas high levels are reported during the resolution phase of inflammation (Gazi and Martinez-Pomares, 2009). CD206 is involved in the internalization of glycoproteins released from neutrophil granules during the resolution phase of wound healing or inflammation. Similar to CD206, Arginase 1 expression is necessary in conversion of L-arginine to ornithine and urea and is implicated as a central pathway of immunosuppression in a range of pathological conditions (Sippel et al., 2015). We found that neutrophils displayed a pro-inflammatory phenotype as evidenced by low levels of CD206 or Arginase 1 at 24 h post-injury. As neutrophils are recruited to the injured brain over the first several weeks post-injury, future studies will determine if these leukocytes maintain a similar phenotype over this more extended period of time.

### 4.5. The Syk kinase signaling pathway as a modifier of anxiety, learning and memory

In the pediatric population, TBI is associated with physical, cognitive, emotional, and social behavioral deficits that may become evident years after the injury (Popernack et al., 2015). Consistent with clinical observations (Narad et al., 2018) and our early studies using wildtype mice (Semple et al., 2015a; Semple et al., 2015b), *syk*^*f/f*^ mice, injured at pnd21, displayed hyperactive behavior at adulthood (Semple et al., 2015a, Semple et al., 2015b). Anxiety disorders have also been reported in children with TBIs (Max et al., 2011). Thigmotaxis in the open field has been validated as a measure of anxiogenic-like behavior in mice (Simon et al., 1994). Rodents typically spend more time exploring the periphery as compared to the more anxiety-provoking center area. Thus, brain injured mice that spend more time exploring the center area demonstrate reduced anxiety-like behavior. Consistent with our previous study, injured mice, irrespective of genotype, displayed reduced anxiety-like behavior, when evaluated in the open field (Tsuru-Aoyagi et al., 2009). However, in the elevated plus maze, injured *syk*^*f/f*^*MRP8-cre*^+^ mice showed reduced anxiety-like behavior as compared to their shams and injured *syk*^*f/f*^ mice. The discrepancy between performance in these tests of the open field and elevated plus maze is not unique to this study and has been reported previously (Rizk et al., 2004; Rochford et al., 1997).

The results of the PCA, showed that percent time spent in the center of the open field and ratio times in the elevated plus maze loaded on different factors, further supporting that these behavioral tests measure different underlying attributes in the mice. In the open field, the mice are started in the center while in the elevated plus maze they are started in an undefined center area between the open and closed arms. Thus, center measures in the open field can be more affected when groups are not matched in activity levels than those in the elevated plus maze. Furthermore, the open field arena is brightly lit, which may lead to different responses (Bailey and Crawley, 2009; Prut and Belzung, 2003). Consistent with our previous studies, we observed genotype-independent changes in increased exploratory and decreased anxiety behavior in injured mice when tested as adults.

Here we report an improvement in performance on the rotarod over time in *syk*^*f/f*^*MRP8-cre*^+^ mice as compared to *syk*^*f/f*^ mice, suggesting an improvement in motor learning. Subsequent posthoc PCA revealed that the total distance moved in the open field and the improvement in rotarod performance loaded on Factor 5. The directions of the component loadings in Factor 5 were such that higher activity levels were associated with improved performance with training on the rotarod. These results reveal that what is measured by assessing motor performance on the rotarod is related to what is measured by assessing motor performance in the open field. Such findings may have relevance to physical therapy to enhance motor performance using either paradigm might generally improve motor function following TBI.

Brain-injured children may show cognitive impairments such as persistent attention, memory and concentration deficits (Anderson et al., 2012). These short- or long-term memory deficits affect new learning and retrieval of previously learned information (Catroppa and Anderson, 2006). Moreover, deficits in executive functioning for behaviors such as inhibition, attentional control, planning, and problem solving are also observed in the pediatric TBI population (Anderson et al., 2013; Babikian et al., 2015; Garcia et al., 2015). These deficits greatly influence academic and work performance and may lead to social dysfunction later in life (Babikian et al., 2015; Ryan et al., 2016). Consistent with our previous studies (Semple et al., 2015b) and studies reported by others (Dixon et al., 1998; Jackson et al., 2018; Whalen et al., 1999), the attenuation of deficits in learning and spatial memory retention in *syk*^*f/f*^*MRP8-cre*^+^ mice did not correlate with long-term sparing of tissue in either the injured cortex or the hippocampus. It is likely that gross volumetric assessments are poor indicators of behavioral metrics because they do not capture the events at the cellular and molecular levels that influence connectivity at the local level as well as between different regions of the brain.

Here, we report attenuation of long-term deficits in spatial learning and memory and thigmotaxis during hidden platform training and probe trials in the MWM test in injured *syk*^*f/f*^*MRP8cre*^+^ mice as compared to *syk*^*f/f*^ mice. There were no TBI-induced or genotype-dependent differences in swim speeds indicating that there were no motor deficits during swimming in response to injury or genotype. Independent of genotype, injured mice showed deficits in task learning during the visible platform trials. Nonetheless, all mice (injured and sham of both genotypes) improved their performance with training during the visible platform trials (task learning). During the hidden platform and probe trials we observed that injured *syk*^*f/f*^*MRP8cre*^+^ mice had improved spatial memory as compared to *syk*^*f/f*^ mice. Our studies offer correlative evidence of decreased neutrophil degranulation, BBB protection and long-term cognitive improvement in *syk*^*f/f*^*MRP8cre*^+^ mice. However, we are encouraged by findings from several studies in the aging brain. In a seminal paper by Zenaro et al. these investigators provided key evidence linking vascular inflammation and a dysfunctional BBB to the pathogenesis of Alzheimer’s disease and cognitive dysfunction. In their study, these authors showed that the LFA-1 integrin controls the movement of neutrophils into the brain and that either neutrophil depletion or blockade of the LFA-1 integrin at the early stages of this disease resulted in reduced pathogenesis and cognitive dysfunction in this disease model (Zenaro et al., 2015). The above findings are further reinforced in studies of the human brain where breakdown of the BBB in the hippocampus was an early predictor of Alzheimer’s related cognitive dysfunction and was independent of Aβ or tau changes (Nation et al., 2019).

On PCA, we observed that cumulative distance to the visible platform in the water maze and percent time spent in the outer zone of the water maze during visible platform training loaded on Factor 3. Increased thigmotaxis in the injured *syk*^*f/f*^ mice may be a consequence of impaired learning and memory that, in turn, results in increased anxiety-like behavior. Alternatively, increased measures of anxiety in the MWM may have contributed to poorer cognitive performance in this maze. Notably, brain-injured animals exhibited genotype-independent anxiolysis in the open field and in the elevated plus maze, brain-injured *syk*^*f/f*^*MRP8cre*^+^ mice showed an increase in time spent in the open arms compared to brain-injured *syk*^*f/f*^ mice and sham controls. Such findings in each of these tests suggest that reduced performance in the MWM may have resulted from deficits in learning and memory rather than anxiety. Cumulative distance to the visible platform in the MWM and percent time spent in the outer zone of the MWM during visible platform training in the MWM loaded on Factor 3. These data indicate that performance during task learning in the MWM (i.e., visible platform training) is unrelated to differences in anxiety-like behavior as assessed in the open field and elevated plus maze.

In the hidden platform trials and probe trials in MWM test, we found reduced spatial learning and memory retention and increased thigmotaxis in injured *syk*^*f/f*^ mice as compared to injured *syk*^*f/f*^*MRP8cre*^+^ mice. An ad hoc PCA was thus used to examine the relationship between these performance assessments. The results of the PCA suggest an anxiety-related component to performance in the MWM test. This is evidenced by greater time spent in the center of the open field (reduced anxiety levels) and in the outer zone of the MWM during hidden platform training as well as during probe trials (increased anxiety levels), and increased cumulative distance to the hidden platform (decreased cognitive function) all loaded onto Factor 1, indicating a close relation between the factors measured by these tests. Mice that spent more time in the center had increased thigmotaxis during hidden platform training and probe trials and swam longer distances in the MWM during hidden platform training. Ratio times in the open arms of the elevated plus maze (negative loading: i.e., increased anxiety levels) and percent time in the target quadrant in the third and fourth probe trials loaded on Factor 2. Mice that showed increased anxiety spent more time in the target quadrant in the third and fourth probe trials. The data on Factors 1 and 2 provide converging evidence for an anxiety-related component to spatial learning and memory-related performance in the MWM test. As the hippocampus is involved in spatial learning and memory and the regulation of anxiety, Factors 1 and 2 might reflect hippocampus-dependent underlying abilities in the mice. Thus, the PCA revealed that measures of anxiety-like behavior contributed significantly to spatial learning and memory in the MWM test. Lastly, although performance in the water maze is hippocampus-dependent and the hippocampus plays an important role in the regulation of anxiety (Bach et al., 2019; Jimenez et al., 2018; Zhu et al., 2019), this does not rule out the possibility that that cortical function is also affected in *syk*^*f/f*^ mice. For example, lesions of the anterior cingulate cortex or dorsomedial striatum impaired abilities to use spatial learning and increased thigmotaxic swimming in the water maze (Pooters et al., 2017). The current study supports the added translational value of considering including a PCA for assessing the effects of TBI on the developing brain as well.

## 5. Conclusion

These studies establish a mechanistic link between the Syk signaling pathway in neutrophil activation/degranulation and long-term neurological recovery after an early age TBI. We show that Syk in neutrophils triggers their activation and conditional deletion of this kinase results in a reduction in ROS in the injured brain, coincident with early neuroprotection and stabilization of the BBB. Importantly, a reduction in early secondary pathogenesis is associated with long-term improvements in learning and memory. From a clinical perspective, SYK specific small molecule inhibitors are a potential target for therapeutic development (Fig. 7). This is exemplified in a recent review by Catz and McLeigh (Catz and McLeish, 2020) who highlighted a new generation of molecular inhibitors that target neutrophil granule trafficking and exocytosis. These inhibitors (Nexinhibs and TAT-peptides, Fig. 7) modulate neutrophil responses through targeting Rab27a/JFC1/SNARE complexes and affecting migration, chemotaxis of immune cells, generation of ROS and release of proteases. Based on our study inhibitors that affect granule exocytosis can serve as therapeutics that will affect neutrophil activation (specifically degranulation, as they act downstream of Syk activation) and can be tested in in vivo models. Thus, there is opportunity to further develop these novel inhibitors to improve cognition in brain-injured children.

## Supplementary Material

Supplemental Figs

## Figures and Tables

**Table 1:** Principal component analysis: component loadings for behavioral measures^a^.

Behavioral measures	Parameters	Factor 1	Factor 2	Factor 3	Factor 4	Factor 5
Open field	Distance moved					0.687
	Time in center	0.720				
Elevated plus maze	Ratio time open arms		−0.625			
Rotarod	Day3-day1					0.716
	Average cumulative distance to the visible platform			0.924		
	Average cumulative distance to the hidden platform	0.737			−0.541	
	% Time in target quadrant in 1st probe trial				0.891	
Water Maze	% Time in target quadrant in 2nd probe trial				0.659	
	% Time in target quadrant in 3rd probe trial		0.697			
	% Time in target quadrant in 4th probe trial		0.858			
	Average % time in the outer zone during visible platform training			0.887		
	Average % time in the outer zone during hidden platform training	0.799				
	Average % time in the outer zone during the probe trial	0.749				

a A principal components factor analysis was performed to determine the relationships between performances on the various tasks on the level of individual mice, and was undertaken to determine whether measures of activity or anxiety-like behavior, across injury conditions and genotypes, might contribute significantly to performance on learning and memory tasks, and to approximate to what extent the distinct learning and memory task measures in the water maze measure the same underlying abilities in the mice. Measures entered into the model were: activity levels in the open field (*Open field (distance moved)*, percentage time in the center of the open-field (*Open Field (time in center)*), ratio times in the open arms of the elevated plus maze (anxiety measure; *Elevated plus maze (ratio time open arms)*), improvement in performance on the rotorod during training (motor learning measure; *Rotarod_(day3-day1)*), mean cumulative to the visible platform location in the water maze (task learning measure; *Water maze (average cumulative distance to the visible platform)*), mean cumulative distance to the hidden platform location in the water maze (spatial learning measure; *Water maze (average cumulative distance to the hidden platform)*), percent time spent in the target quadrant in the first, second, third, and fourth probe trials (spatial memory retention measures; *Water maze (percent time in target quadrant in first, second, third, or fourth probe trial)*), average percent time spent in the outer zone of the water maze during visible platform training (anxiety measure; *Water maze (average percent time in the outer zone during visible platform training)*), average percent time spent in the outer zone of the water maze during hidden platform training (anxiety measure; *Water maze (average percent time in the outer zone during hidden platform training)*), average percent time spent in the outer zone of the water maze during the four probe trials (anxiety measure; *Water maze (average percent time in the outer zone during the probe trials)*). The verimax rotated matrix was used to interpret the factor loadings. This analysis revealed five factors with eigenvalues >1.0, which explained a total of 74.4% of the variance among the measures entered into the model (Table 1). The five factors explained 20.9, 15.0, 14.7, 14.5, and 9.4% of the variance, respectively. For additional details, see main text.

## Abbreviations:

BBB: Blood-brain barrier
DG: Dentate gyrus
EBA: Evans blue albumin
MWM: Morris water maze
NE: Neutrophil elastase
p21: Postnatal day 21
PCA: Principal components factor analysis
ROS: Reactive oxygen species
Src: tyrosine kinase
Syk: Spleen tyrosine kinase
TBI: Traumatic brain injury
