# Supplementary material for: Neutrophil-specific deletion of Syk results in recruitment-independent stabilization of the barrier and a long-term improvement in cognitive function after traumatic injury to the developing brain
Source: Neurobiol Dis. Author manuscript; Available in PMC 2024 Aug 6. (PMC11302380; doi:10.1016/j.nbd.2021.105430)
Supplement: Supplemental Figs [file NIHMS2005555-supplement-Supplemental_Figs.pdf]

SUPPLEMENTAL MATERIAL

Neutrophil-specific deletion of Syk results in recruitment-independent stabilization of the barrier and a long-term improvement in cognitive function after traumatic injury to the developing brain.

Alpa Trivedi, Kayleen G Tercovich, Amy Jo Casbon, Jacob Raber, Clifford Lowell, Linda J Noble-Haeusslein

Neurobiol Dis. 2021 Sep:157:105430. doi: 10.1016/j.nbd.2021.105430. Epub 2021 Jun 19.

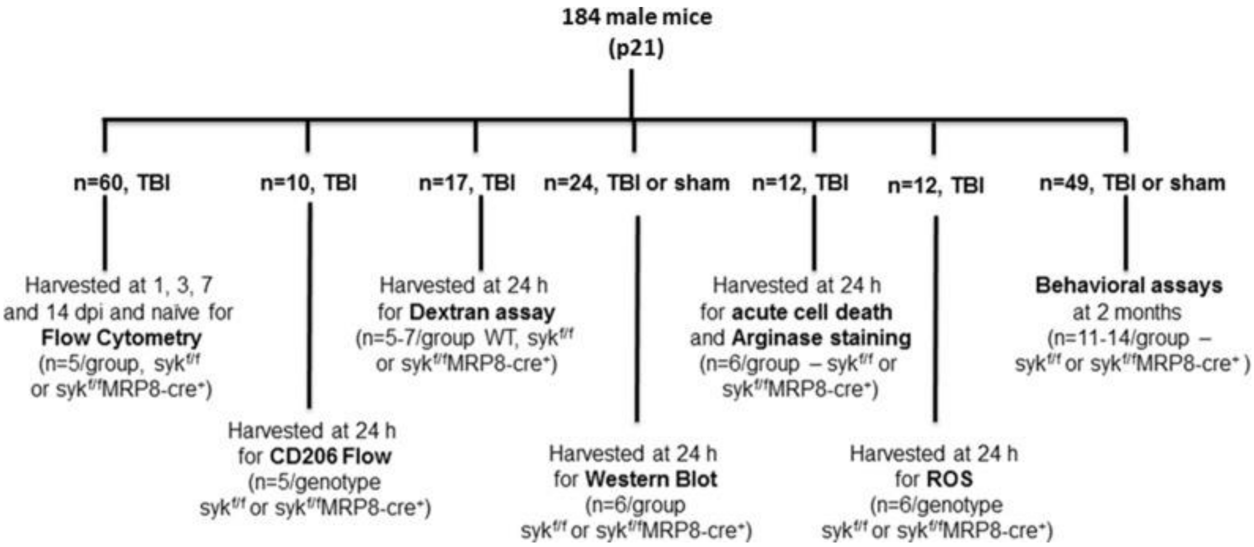

1. Download: Download high-res image (175KB)
2. Download: Download full-size image

Fig. 1. Experimental design summarizing all animals enrolled in the acute and long-term experiments. Note that there was no mortality in this study. Animals underwent randomization for injury allocation, and injured animals were randomized for long-term behavioral assays. All experiments were conducted blinded to genotype.

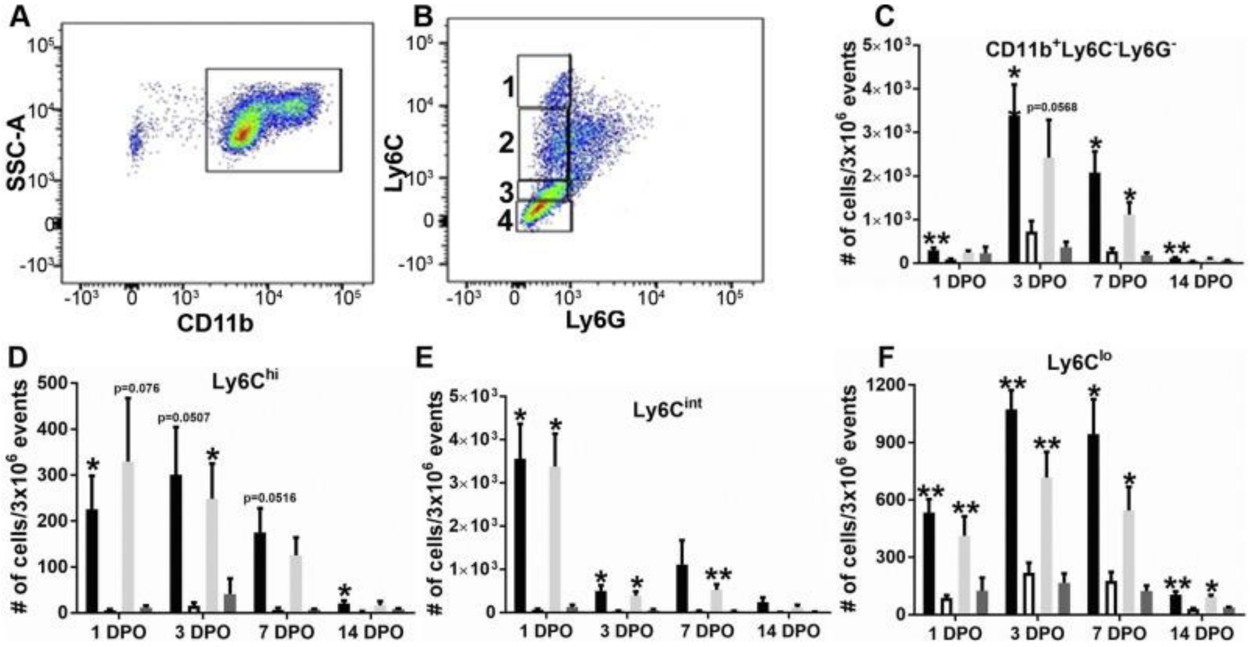

1. Download: Download high-res image (324KB)
2. Download: Download full-size image

Supplemental Fig. 2. There is a prolonged recruitment of monocyte/macrophages into the brain over the first 14 days post-injury (DPO) that is independent of genotype.
A, B). Gating was used to identify monocyte subtypes ($CD11b^+$ and $Ly6C^+$ cells) 1. $Ly6C^{hi}Ly6G^-$ (pro-inflammatory monocytes); 2. $Ly6C^{int}Ly6G^-$ (anti-inflammatory monocytes); 3. $Ly6C^{lo}Ly6G^-$ (resident/patrolling macrophages); 4. $CD11b^+Ly6C^-Ly6G^-$ (undifferentiated myeloid cells, microglia, dendritic cells). C–F) There are no genotypic differences in the temporal recruitment of leukocytes [$CD11b^+Ly6G^-Ly6C^-$ (C)] into the injured hemisphere. Similar findings were seen with subsets monocytes [$Ly6C^{hi}$ (D), $Ly6C^{int}$ (E) and $Ly6C^{lo}$ (F). Bars represent mean + sem. Comparisons are between the ipsilateral and contralateral hemispheres (paired two-tailed t-test). As indicated on graph, *$p < 0.05$; **$p < 0.01$; ***$p < 0.001$; n = 5 mice/genotype/time-point. Comparisons between the ipsilateral hemispheres of the two genotypes (unpaired two-tailed *t*-test). All comparisons had $p > 0.05$.

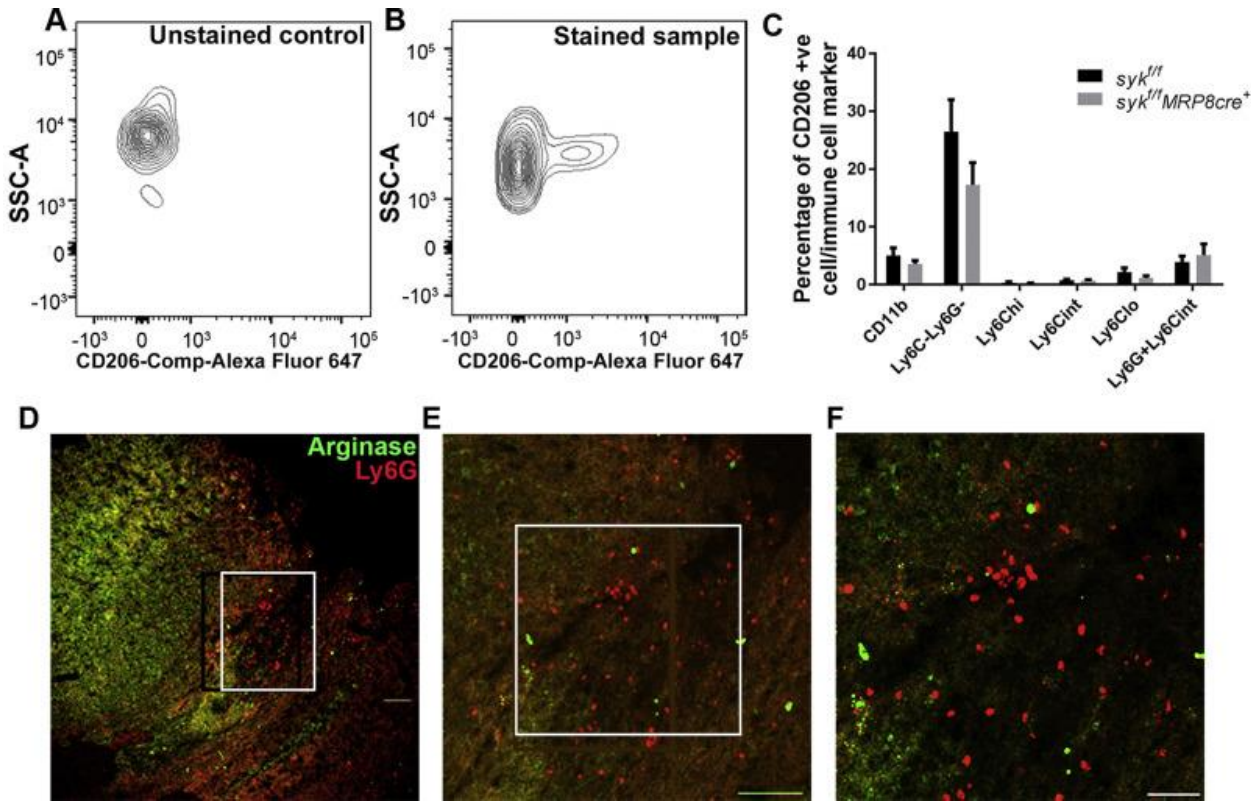

1. Download: Download high-res image (661KB)
2. Download: Download full-size image

Supplemental Fig. 3. There are no differences in the expression of the anti-inflammatory markers, CD206 and arginase in brain-injured *syk*$^{f/f}$ versus *syk*$^{f/f}$*MRP8cre*$^{+}$ mice at 24 h post-injury.

A) Representative flow cytometry tracing and CD206 gate in the unstained brain (control) digests. B) Representative flow cytometry tracing in tissue digest stained with the CD206 antibody. C) There is modest to barely detectible CD206^{+} staining in monocyte populations (Ly6C^{hi}, Ly6C^{int}, Ly6C^{lo}). In contrast, CD206 is expressed at low levels in CD11b^{+}, and in Ly6G^{+}Ly6C^{int} cells in both genotypes, but at highest levels in Ly6C^{-} Ly6G^{-} cells There is no difference in CD206 staining between genotypes (unpaired two tailed t-test, CD11b^{+}, $p = 0.3139$; Ly6C^{-} Ly6G^{-}, $p = 0.1884$; Ly6G^{+}Ly6C^{int}, $p = 0.9648$; Ly6C^{hi}, $p = 0.5381$; Ly6C^{int}, $p = 0.8491$; Ly6C^{lo}, $p = 0.1835$). Bars represent mean + sem. n = 7 *syk*$^{f/f}$; $n = 8$ *syk*$^{f/f}$*MRP8cre*$^{+}$. D—F) Arginase 1 does not co-immunolocalize with Ly6G^{+} neutrophils in the cortex near the site of injury at 24 h post-injury. A representative section immunolabeled for arginase 1 (green) and Ly6G (red), (D). Higher magnifications shown in (E) and (F). Scale bars: 100 µm.

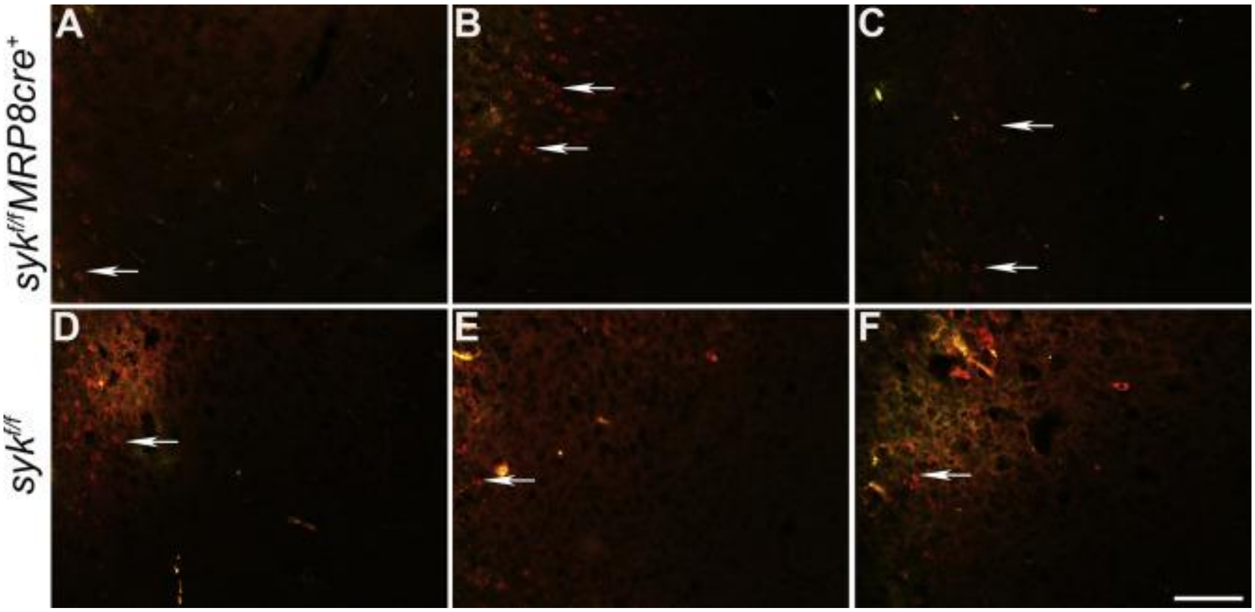

1. Download: Download high-res image (281KB)
2. Download: Download full-size image

Supplemental Fig. 4. More tissue leakage to dextran dyes in injured *syk*$^{f/f}$ mice as compared to *syk*$^{f/f}$*MRP8cre*$^{+}$. In both genotypes, there is evidence of TMR-labeled neurons (arrows) in proximity to the injured zone which likely reflects dying/dead neurons and nonspecific uptake of the tracer. In contrast, the key distinguishing factor between genotypes is the diffuse and more pronounced pattern of acellular TMR and FITC-labelling that appears more prominent in the *syk*$^{f/f}$ (D–F) compared to the *syk*$^{f/f}$*MRP8cre*$^{+}$ (A-C) animals (three individual animals per genotype are represented). Scale bar is F = 100 μm.

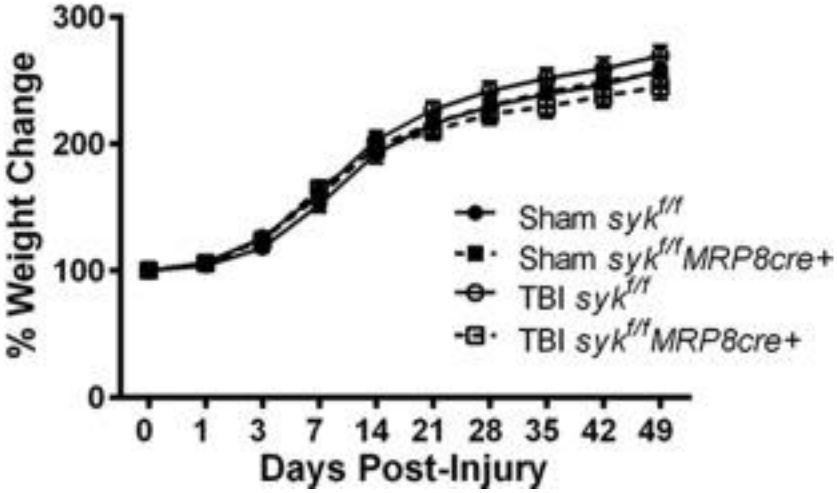

1. Download: Download high-res image (104KB)
2. Download: Download full-size image

Supplemental Fig. 5. Overall health was assessed by weight gain over time in each of the genotypes. All injured and sham animals had similar weight gain over time, with no effect of genotype (RM two way ANOVA, no significant interaction, effect of injury/genotype (ns), effect of time (p < 0.0001)).

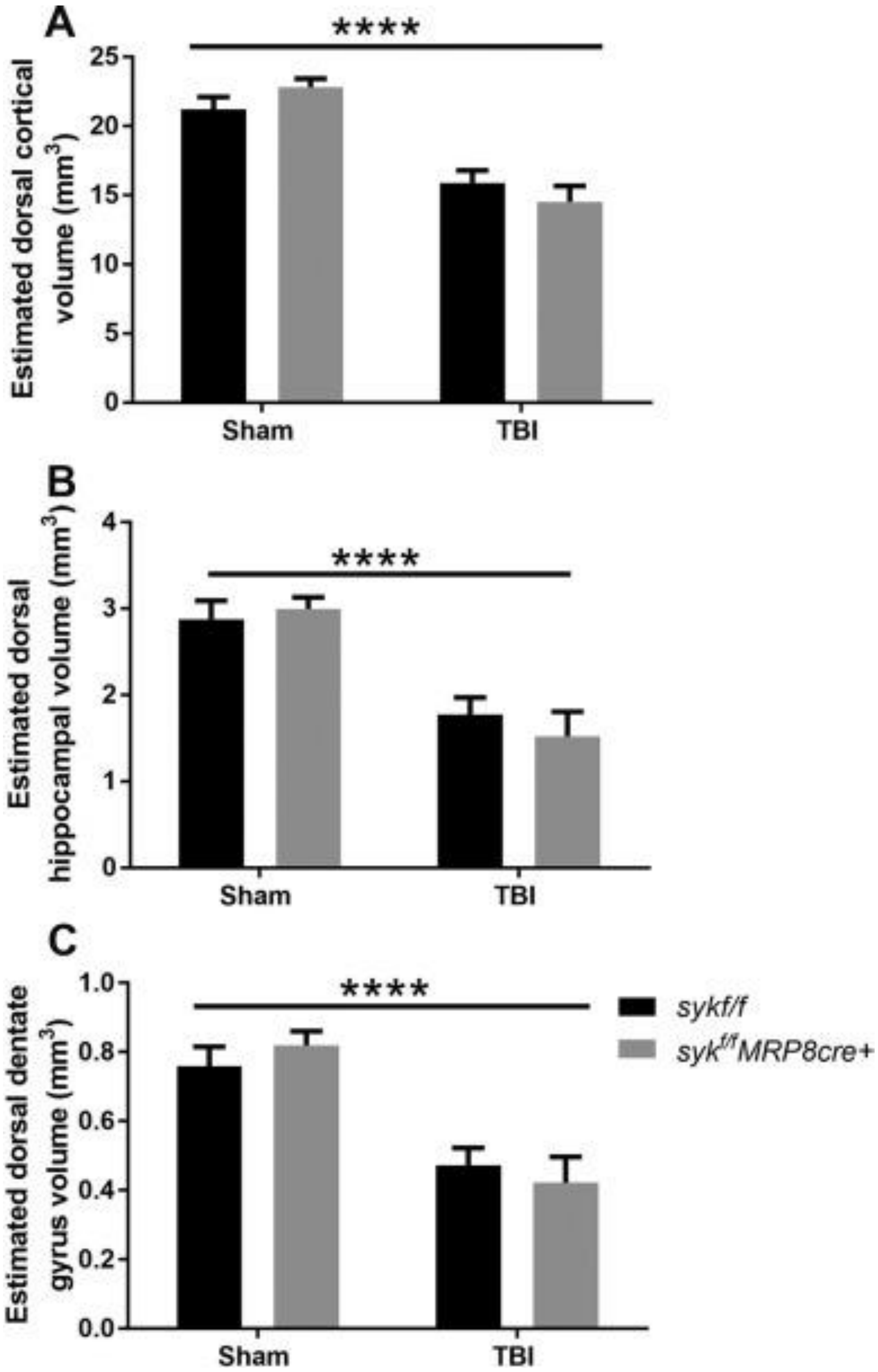

1. Download: Download high-res image (137KB)
2. Download: Download full-size image

Supplemental Fig. 6. Conditional Syk gene deletion does not alter long-term volumetric loss in the ipsilateral cortex and hippocampus. Stereological analysis was performed on Cresyl violet stained sections in the ipsilateral dorsal cortex and hippocampus at 3 months of age (~2 months post-injury). A) Quantification of the ipsilateral dorsal cortex revealed a reduction in cortical volume in brain-injured mice compared to sham controls (2-way ANOVA effect of injury $F(1, 42) = 53.35$, $p < 0.0001$), with no differences between genotypes (effect of genotype, $F(1, 42) = 0.0128$, $p = 0.9104$; injury x genotype interaction $F(1, 42) = 2.564$, $p = 0.1168$). B) The ipsilateral hippocampal volume was reduced (2-way ANOVA effect of injury $F(1, 43) = 33.14$, $p < 0.0001$), and there was no effect of genotype (effect of genotype $F(1, 43) = 0.08303$, $p = 0.7746$; injury x genotype interaction F1, 42 = 0.6743, $p = 0.4161$). C) The ipsilateral dentate gyrus showed an effect of injury induced volumetric loss (2-way ANOVA effect of injury $F(1, 43) = 33.52$, $p < 0.0001$), with no effect of genotype (effect of genotype $F(1, 43) = 0.0062$, $p = 0.9375$; injury x genotype interaction $F(1, 43) = 0.8641$, $p = 0.3578$). $n = 11$–13/group.

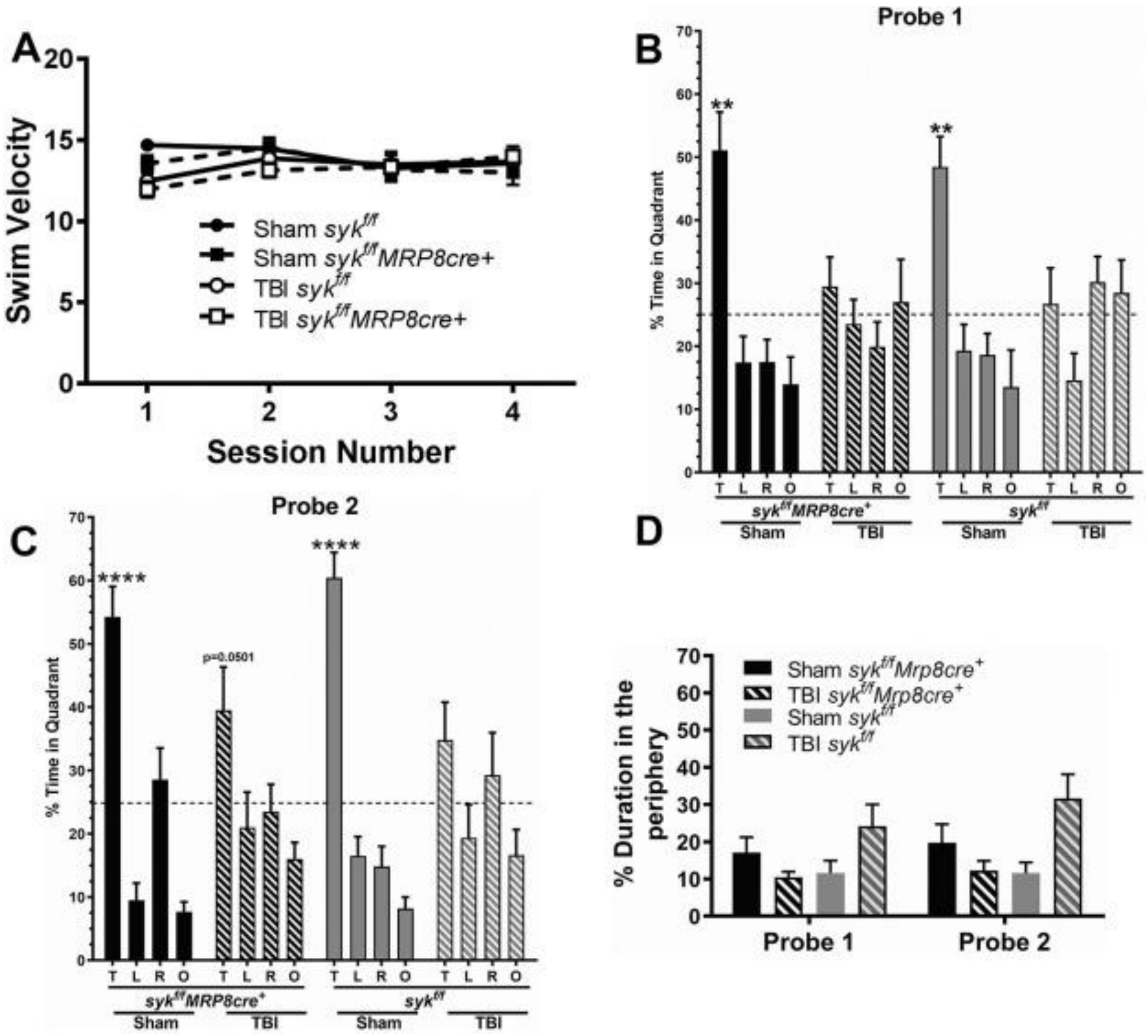

1. Download: Download high-res image (368KB)
2. Download: Download full-size image

Supplemental Fig. 7. As part of the MWM paradigm, swim velocities were evaluated prior to assessment of performance in the hidden platform sessions. Additionally, probe trials were conducted to assess memory retention after the each of the hidden platform sessions.
A) All mice had similar swim velocities irrespective of injury or genotype (RM two-way ANOVA, effect of injury ns, effect of genotype ns) n = 11–13/group. B) At the end of the first hidden platform session, probe trials revealed that sham controls, representing both genotypes, spent more time in the target quadrant (T) compared to other quadrants (L-left, R-right, O-opposite). In contrast, brain-injured mice showed no preference for the target quadrant (RM one way ANOVA with Dunnett's test, statistics as shown on graph *$p < 0.05$, **$p < 0.01$) n = 11–13/group. C) At the end of the second hidden platform session, another probe trial was conducted. Sham controls, representing both genotypes, spent more time in the target quadrant

(T) compared to other quadrants (L-left, R-right, O-opposite), whereas brain-injured mice showed no preference (RM one way ANOVA with Dunnett's test, statistics as shown on graph ****$p < 0.0001$). Interestingly, the injured *syk*$^{f/f}$*MRP8cre*+ mice had a strong tendency to spend more time in the target quadrant as compared to the opposite quadrant (RM one way ANOVA with Dunnett's test, $p = 0.0501$) n = 11–13/group. D) When thigmotaxis was analyzed during the probe trials, there was an effect of TBI ($F = 4.451$, $p = 0.018$) and a genotype x TBI interaction ($F = 10.064$, $p < 0.001$). There was an effect of TBI in *syk*$^{f/f}$ ($F = 12.740$, $p < 0.001$) with greater thigmotaxis in TBI than sham mice but not in *syk*$^{f/f}$*MRP8-cre*$^{+}$mice ($F = 0.743$, $p = 0.487$). Values are mean + sem.
